# Is Working Risky or Protective for Married Adolescent Girls in Urban Slums in Kenya? Understanding the Association between Working Status, Savings and Intimate-Partner Violence

**DOI:** 10.1371/journal.pone.0155988

**Published:** 2016-05-27

**Authors:** Eunice Muthengi, Tabither Gitau, Karen Austrian

**Affiliations:** Poverty Gender and Youth Program, Population Council, Nairobi, Kenya; The Hospital for Sick Children, CANADA

## Abstract

**Introduction:**

Previous studies have shown that women’s empowerment, though beneficial in many aspects, can also increase the risk of intimate-partner violence (IPV). This study seeks to examine the association between work and experience of physical violence among married adolescents, and to understand the impact of access to independent financial resources on this risk. Authors draw on the asset-building framework and the ecological model.

**Methods:**

The data is from a baseline survey of girls aged 15–19 residing in urban slums in four cities and towns in Kenya (Nairobi, Thika, Nakuru and Kisumu). The analytic sample is 452 married girls. Logistic regression is used to examine associations between working status, savings and experience of IPV in the previous six months, controlling for other factors. This is complemented by content analysis of in-depth interviews with 32 adolescent girls and 16 young men.

**Results:**

Compared to girls who did not work, working with no regular savings was significantly associated with greater odds (OR = 1.96, p<0.01) of experiencing IPV. There was no difference between girls who did not work and those who worked but had regular savings. Qualitative findings indicate savings decrease girls’ dependency on men and allow them to leave abusive partners.

**Discussion:**

Findings imply that in these communities with patriarchal gender norms and high levels of poverty, female employment and financial conflicts can be triggers of violence in marriages. On the other hand, girls’ management of and access to independent financial resources through savings can potentially help to reduce this risk.

## Introduction

Intimate-partner violence (IPV), recognized as a human rights violation, is one of the most common forms of gender-based violence. Approximately 39% of women in East Africa have ever experienced intimate partner violence [1]. In the 2014 Kenya Demographic and Health Survey, the prevalence of physical violence among ever-married women was 38% for all women and 24% of girls aged 15–19. About one in five young women (ages 15–19) had experienced physical violence committed by their husband or partner within the past year [2]. Physical violence was defined as being harmed by someone pushing, slapping, punching, kicking or trying to strangle or burn them or threaten them with a weapon.

Consequences of IPV include adverse health outcomes, including physical, mental and reproductive health. According to a WHO conceptual framework, the hypothesized pathways for these effects include physiological, behavioral and indirect effects through stress responses [1]. Physical trauma causes direct injuries which are associated with disability and death. Psychological trauma and stress can lead to substance abuse and mental health problems such as anxiety, PTSD, depression, and suicidality. These effects are associated with increased risk of non-communicable diseases and mental illnesses that cause bodily symptoms. The fear and control associated with physical violence can limit sexual and reproductive control and health seeking behaviors, resulting in increased risk of perinatal and maternal complications, unwanted pregnancy, sexually transmitted infections and abortion.

In addition to the public health impact and health care costs, violence against women has significant economic costs to women, their families and societies at large. Intimate partner violence has been linked to reduced education for women and their children, income loss, reduced productivity and loss of human capital, all of which have implications for economic growth [3]. However, the relationship between IPV and work for women is complex, as some studies indicate that work is protective while others indicate that women who are employed are more likely to experience violence.

In a study using Demographic Health Surveys (DHS) data from 10 countries, not working was protective in Bolivia, the Dominican Republic and Zimbabwe, while working in agriculture was a risk factor in Malawi [4]. A review of the literature exploring the relationship between women’s economic empowerment and risk of IPV also highlights the complexities that have resulted in mixed findings [5]. In studies in India, rural Bangladesh, Nicaragua and Dominican Republic, employment was associated with increased risk [6, 7]. In Haiti, urban Bangladesh, Zambia and Cambodia there was no association, but in Haiti and Egypt earned income was protective against physical violence. In urban India, although women who worked had a higher prevalence of physical violence, they were more likely to seek help for IPV [8]. A randomized controlled trial in Ethiopia showed that employment opportunities may increase risk of violence because men feel “threatened” by the autonomy earned by their wives [9].

There is some evidence that empowerment programs can reduce risk of IPV, especially when combined with social empowerment approaches. An evaluation of the IMAGE study in South Africa, a women’s micro-credit program, showed a reduction in the risk of experiencing violence due to both women’s economic and social empowerment [10]. A study in northern India found protective effects of financial autonomy and freedom of movement in reducing the risk of marital violence [11]. Similarly, a randomized-controlled trial in Cote d’Ivoire showed a greater decrease in reports of IPV for women who participated in group savings in addition to gender dialogue group, as compared to group savings alone [12]. In one of the few studies of adolescents, an evaluation of a program aimed at building girls’ social health and economic assets in Uganda, researchers found an increased risk of sexual harassment for girls who received individual savings accounts alone, compared to those who also participated in safe spaces groups where they received financial education and reproductive health training [13].

Economic empowerment of vulnerable girls is a recommended strategy for preventing the risk of gender-based violence, particularly as part of a multi-sectoral approach [14, 15]. However, as the literature on women’s programs shows, understanding the potential risks of economic strengthening is key to minimizing harm to girls. This study makes a unique and significant contribution to the wide literature on empowerment and IPV because it is one of the first to demonstrate the association between work, savings and IPV among adolescent girls. Furthermore, the study focuses on a sample of vulnerable girls who are disadvantaged both due to early marriage and residence in urban slums. Using both quantitative and qualitative methods, we seek to examine the association between work and experience of physical violence, and to understand the impact of access to independent financial resources (savings) on this risk.

Authors draw on the ecological model and the asset-building framework to hypothesize about the relationships between work, savings and IPV. The ecological model recognizes that there are multiple causes of violence that operate through individual, microsystem (situational), exosystem and macrosystem levels [16]. Individual level factors include witnessing marital violence as a child, or being a survivor of abuse. At the microsystem level, factors such as male dominance, male control over wealth, use of alcohol and marital conflict can increase risk of IPV. Exosystem factors include low socioeconomic status, unemployment, isolation of women, and delinquent peer associations. At the macrosystem level, male entitlement can exacerbate violence, as well as masculinity linked to dominance, rigid gender roles, acceptance of interpersonal violence and acceptance of physical chastisement.

The asset-building framework posits that building girls’ economic assets in combination with building their health and social assets has the potential to decrease their exposure to violence and exploitation by enabling them to gain more control over their lives, particularly decisions regarding their sexual health and relationships [17, 18]. Assets, such as savings, can be viewed as a store of value that girls can use to both reduce vulnerabilities and expand opportunities. Savings help girls to mitigate the economic challenges that they face in their daily lives by serving as a safety net for emergencies, providing capital for future events and enabling them to make healthier decisions. Studies have shown a positive association between economic strengthening and improvements in girls’ sexual behavior, as well as other reproductive health outcomes [19, 20].

A qualitative study of girls residing in informal settlements in Kenya showed that they are often financially dependent on men and therefore lack decision making power in their sexual relationships [21]. Girls reported that earning money, saving money and having control over money allows them to be less dependent on men and therefore have more decision making power in their sexual relationships.

**Fig 1:**
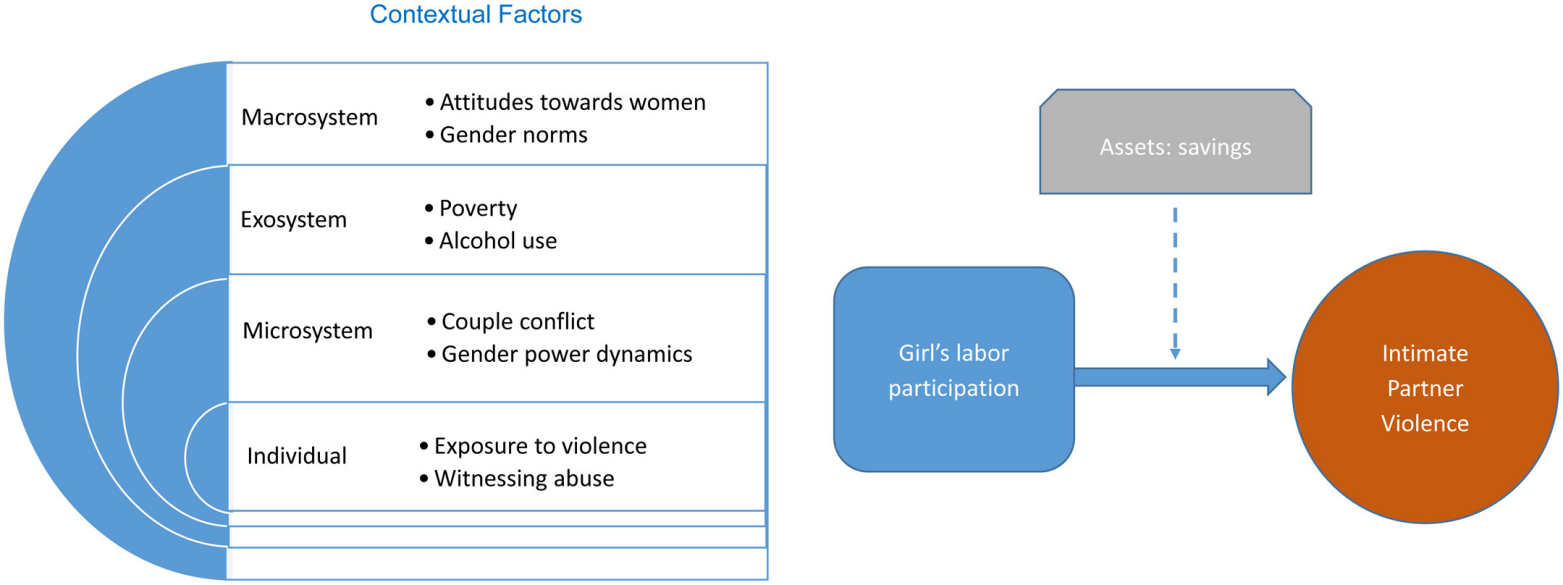
Conceptual Framework Based on Ecological Model and Asset Building Framework.

As shown in the conceptual framework (Fig 1), authors hypothesize that working girls in Kenyan urban slums might be at greater risk of IPV due to the poverty experienced in urban slums, greater exposure to exploitation and increased interactions with males in a male-dominated society. Although these dynamics can increase girls’ risk of IPV, economic assets such as savings can decrease their dependency on men and therefore enable them to avoid potentially violent situations. In addition, economic assets can provide girls with more alternatives for how to react if they experience violence. As financial issues and control over money can increase tension within a marriage for working girls, having increased trust regarding money management in the relationship, could reduce the risk of IPV.

## Materials and Methods

### Quantitative Study

#### Data

This study uses a dataset of adolescent girls residing in low-income, informal settlements (slums) in four Kenyan cities and towns: Nairobi, Kisumu, Nakuru and Thika. The data were collected between August and December, 2013 as part of a baseline survey for an intervention program aimed at building social, health and economic assets for vulnerable adolescent girls in the four cities locations. The four sites were selected because they had large urban slum populations and access to the financial institution partnering on the interventions. Based on consultations with implementing partners, two equivalent sites in each city/town were selected, one as the project site and one as the comparison site. An initial household listing took place in each site, listing all members of all households to identify adolescent girls. Eligibility was based only on age and residence in study sites. All girls between the ages of 15 and 19 were invited to take part in the study, and interviewed using a structured questionnaire programmed onto tablets. If the respondent was not present at the household on the day of the interviewer visit, the interviewer paid up to three visits to the household in order to locate and interview the selected respondent, similar to the procedure used in the DHS. Written informed consent/assent was obtained from all respondents before conducting interviews. Parental consent was obtained for all respondents below the age of 18 who were unmarried. Married girls were considered to be emancipated minors and able to provide consent. Interviews were conducted face to face in a private area, with visual and auditory privacy.

The Economic Assets and GBV Baseline Survey [S1 File] covered a broad range of issues, but focused on: knowledge, attitudes and practices related to saving, financial education, livelihoods, and reproductive health behavior, sexual behavior, sexual harassment and experience of sexual violence. The survey was translated into Swahili, pilot-tested and revised before data collection began. Ethical approvals were obtained from the Population Council Institutional Review Board and the Kenyatta National Hospital/University of Nairobi Ethical Review Committee. Written informed consent was obtained prior to all data collection activities by the interviewer in a private setting. Following established informed consent protocols, each respondent was provided a thorough explanation of the purpose of the study, the privacy and confidentiality of their responses, and the process and extent of participation. For respondents under 18 years of age, written informed consent from a parent or guardian was obtained prior to the interview. Written assent from the girls was also obtained separately for the interview. For those 18 and older, informed consent for the interview was obtained only from the girl herself.

A total of 28,768 households were listed, 5,100 eligible girls were identified, and 3,255 interviews were conducted, with a response rate of 64%. The main reasons for non-response were refusal to consent to the interview, and inability to locate the respondent after three visits. After excluding 31 girls who were outside the age range of 15 to 19, the total sample included 3,224 interviews girls. The analytical sample for this study was 452 married girls who were living with their husbands at the time of the survey.

#### Measures

Physical violence was based on a question asking: “How many times in the last six months has your husband/partner hit, slapped, kicked or beaten you?” A dichotomous variable was created by recoding this variable to compare those who responded “0” and those who responded 1 or more.

Work status was based on a question asking whether they had done various work activities in the past one year, including: domestic work, washing clothes only, washing utensils only, washing house only, fetching water, plaiting hair/beauty, babysitting/childcare, work in a hotel/restaurant, work in a farm, packaging food or drinks, selling things, work in an office, other types of own business, other types of temporary jobs, and other types of formal employment. For each activity, girls responded yes or no, and if yes, they were asked about the amount earned per month, the number of months per year that they earned the income and whether they were working as hired staff, a temporary job or a running a business.

Girls were asked whether they had saved any money within the previous six months. Of those who saved, girls were further categorized as regular or irregular savers. Regular savers were those who answered either “always” or “usually” instead of “sometimes” or “never” to the following question: “Would you say that you saved on a weekly basis always, usually, sometimes or never?”

Partner trust was a dichotomous variable indicating girls who agreed or disagreed with the statement: “Your partner trusts you with money”

#### Analysis

Bivariate analysis was conducted to compare demographic characteristics between girls who worked for pay within the previous year as well as to examine differences in experience of physical violence by work and savings characteristics among girls who did work. The Pearson’s Chi-Square test was used to test for significant differences at the level of p<0.05. Multivariate logistic regression models were used to estimate the effect of work status, saving, and trust regarding money on the experience of physical violence adjusting for demographic and other factors. In bivariate and multivariate analysis, the survey design is taken into account with Stata’s survey analysis technique to account for clustering by site and treatment arm.

### Qualitative Study

The qualitative data was from a larger qualitative study implemented to provide an in-depth understanding of the relationship between economic assets and exposure to sexual violence and exploitation, as well as an assessment of the strategies that can mitigate that exposure while girls continue to increase their economic independence. For this paper, only responses related to intimate-partner violence were analyzed and summarized. As no differences were observed in the perceptions of non-married respondents (cohabiting or in relationships) as compared to married adolescents, all relevant responses were included to provide a better understanding of relationship dynamics in these contexts.

In-depth interviews were conducted between July and August, 2014 with adolescent girls between the ages of 15 to 24 and young men between the ages of 20 and 30, who were more likely to be engaged in exploitive sexual relationships with girls of this age. Purposive samples of adolescent girls and young men were recruited in collaboration with identified implementing partners and local leadership. The female and male samples were recruited from two different villages within study sites, to ensure that the men interviewed would have no connections or relationships to girls interviewed. Each respondent was visited over two to three rounds of interviewing, progressively covering more sensitive topics on successive visits. This technique is valuable in promoting candor during the interview process, particularly on sensitive topics.

Eligible participants will be selected based on target profiles stratified by age, schooling status and geographical site (Table 1). A total of 48 individuals were interviewed, 12 in each site, including: 16 adolescent girls 15–19, 16 adolescent girls 20–24 and 16 young men 20–30.

**Table 1:** Number of In-Depth Interview Participants, by Category and Site.

	Site			
	Kariobangi	Nakuru	Thika	Kisumu
In-school adolescent girls 15–19	2	2	2	2
Out-of-school adolescent girls 15–19	2	2	2	2
Out-of-school adolescent girls 20–24	4	4	4	4
Young men 20–25	2	2	2	2
Young men 25–30	2	2	2	2
**Total**	**48**			

The In-Depth Interview Guide for Adolescent Girls [S2 File] and the In-Depth Interview Guide for Young Men [S3 File] included suggested topic areas as well as possible accompanying probes. The interview guides were translated into Swahili, pilot-tested and revised before data collection began. Once participants are identified, interviewers visited them in their homes, reviewed the consent forms and conducted interviews in a private area, with visual and auditory privacy. All interviews were tape recorded, transcribed and translated from Swahili into English. Using Atlas.ti. software, a codebook was developed and transcripts were coded and reconciled by two coders. Using an inductive thematic analysis approach [22], data were analyzed to identify core theoretical concepts, themes and patterns.

## Results

### Quantitative Results

Married girls who had worked did not significantly differ from married girls who had not worked on most demographic characteristics (Table 2). Half of the girls were age 19 (55%), a third were age 18 (33%) and 12% were between the ages of 15 and 17. The mean age of marriage was 17 years and the mean husband’s age at the time of the survey was 24 years. Almost all girls reported that their husbands had worked for pay during the previous month (91%). The majority of girls were Christian (70% Protestant, 25% Catholic).

**Table 2:** Demographic Characteristics by Work Status, Currently Married Girls, Age 15–19.

Key Characteristics	Work Status		
	Did Not Work	Worked in Past Year	Total
	(N = 257)	(N = 195)	(N = 452)
**Age:**			
15	†0.0%	1.0%	0.4%
16	2.7%	1.5%	2.2%
17	9.0%	8.7%	8.9%
18	37.0%	28.7%	33.4%
19	51.4%	60.0%	55.1%
**Mean Husband’s Age**	24.15	24.34	24.23
**Mean Age at First Marriage**	17.27	17.35	17.30
**Husband Worked Prev Month:**			
Yes	91.8%	90.3%	91.2%
**Religion:**			
Catholic	24.5%	26.2%	25.2%
Protestant	70.0%	68.7%	69.5%
Other	5.5%	5.1%	5.3%
**Orphan-hood:**			
Mother Deceased	6.2%	13.9%	9.5%
Father Deceased	23.4%	18.0%	21.0%
Both Deceased	13.2%	13.3%	13.3%
Both Alive	57.2%	54.9%	56.2%
**Number of Children:**			
None	37.4%	42.1%	39.4%
1	51.4%	50.3%	50.9%
2+	11.3%	7.7%	9.7%
**School Status:**			
Currently in school	*0.4%	3.0%	1.6%
**Educational Attainment:**			
Primary (1–7)	25.8%	26.0%	25.9%
Primary Complete	30.5%	38.5%	33.9%
Some Sec./Vocational	30.7%	24.0%	27.9%
Secondary Complete	12.9%	11.5%	12.3%
**Water Source:**			
Piped into Residence	40.0%	43.1%	41.4%
Piped Water-Bought	52.5%	53.3%	52.9%
Piped Water-Free	5.8%	3.1%	4.7%
Other	1.6%	0.5%	1.1%
**Household Assets:**			
Electricity	67.7%	62.1%	65.3%
Telephone/Mobile	85.6%	92.3%	88.5%
Jewelry	31.5%	35.9%	33.4%
**Girl’s Assets:**			
Telephone/Mobile	50.2%	60.0%	54.4%
Jewelry	29.6%	34.9%	31.9%
**City:**			
Thika	24.1%	17.4%	21.2%
Nakuru	6.6%	8.2%	7.3%
Kisumu	40.1%	33.3%	37.2%
Kariobangi	29.2%	41.0%	34.3%

*p < .05;

^†^<0.10

About one in ten married girls had lost their mother (10%), and one out of five had lost a father (21%) and 13% had lost both parents. Most girls had begun childbearing with 51% having one child and 10% having at least two children. Household living conditions and ownership of assets did not differ between working and non-working girls. About half of girls (54%) owned a mobile phone, 32% owned jewelry, 65% resided in a home with electricity, and less than half had water piped to their residence (41%). The only significant difference between girls who worked and those who did not work was their current school status. Almost all girls had dropped out of school by the time of the survey (98%), but girls who worked were slightly more likely to be in school (3%) than those who did not (0.4%).

About two-fifths (43%) of girls had worked for pay within the previous year. The mean age at which they first started working for pay was 16.6 years. The types of work included: domestic worker (32%), temporarily doing housework or childcare (26%), working in a hotel (25%), selling things (24%) plaiting hair (9%) and other work (15%). Most girls did only one type of job (67%), one in five did 2 jobs (22%) and 12% of girls did 3 or more jobs. Almost half of girls (45%) reported working for at least 7 months of the year. The mean monthly income from all jobs was Ksh 3,731 (USD 43.9). More than half of girls (59%) did not save any of their money within the previous six months. A quarter of girls saved irregularly (26%) and 16% saved usually or always. Most girls reported that their partner trusts them with money (88%).

As shown in Table 3, at the bivariate level, about a quarter of girls who worked experienced physical violence in the previous 6 months compared to 16% of girls who did not work (p<0.05). Of the working characteristics described above, the only one associated with violence was the partner’s trust with money. More than half (60%) of girls whose partners did not trust them with money experienced violence, compared to 21% of those who did (p<0.05).

**Table 3:** Description of Work Characteristics and Experience of Physical Violence, Married Girls who Worked in Past year, Age 15–19 (N = 195).

Selected Characteristics	Frequencies Among Girls Who Worked	% Experienced Physical Violence in Last 6 Months
Did not work		*16.0%
Worked in Previous year		25.6%
**Mean Age First Started Working for Pay**	16.61	
**Type of Job**		
Housework/childcare	25.6%	40.0%
Domestic worker	32.3%	34.9%
Hotel	24.6%	33.3%
Selling things	24.1%	19.2%
Plaiting hair	9.2%	11.1%
Other	14.9%	17.2%
**Mean Monthly Income**	3,731 KES (USD 43.9)	
**Total Monthly Income:**		
76 KES (USD 0.9)– 1,501 KES (USD 17.7)	30.3%	20.3%
1,503 KES– 3,001 KES (USD 35.4)	22.6%	25.0%
3,005 KES– 5,001 KES (USD 58.8)	26.2%	29.4%
5,002 KES– 20,001 KES (USD 235.3)	21.0%	29.3%
**Number of jobs:**		
1	66.7%	19.2%
2	21.5%	33.3%
3+	11.8%	47.8%
**Number of Months Worked:**		
1–3	36.9%	22.2%
4–6	18.0%	25.7%
7–12	45.1%	28.4%
**Saved in Past Six Months:**		
No	58.5%	28.1%
Saved Sometimes/Never	25.6%	22.0%
Saved Usually/Always	15.9%	22.6%
**Partner Trusts with Money:**		
No	11.8%	*60.9%
Yes	88.2%	20.9%

*p < .05

Multivariate logistic regression models were estimated to examine the effect of the key variables (work status, saving and partner trust) on experience of violence, controlling for age, education, religion, socioeconomic status (ownership of jewelry), husband’s age, husband’s work status and number of children (Table 4). Of these, factors associated with a reduced odds of experiencing physical violence were primary education (OR = 0.400, p<0.05), secondary education (OR = 0.209, p<0.01), and ownership of jewelry (OR = 0.549, p<0.05).

**Table 4:** Multivariate Logistic Regression Results Predicting Experience of Physical Violence in the Previous Six Months, Married Girls, Age 15–19 (N = 445).

	Model 1: Original Model		Model 2: Work Status and Saving Status Interaction		Model 3: High/Low Work Income and Saving Interaction	
	OR	[95% CI]	OR	[95% CI]	OR	[95% CI]
**Age (Ref = 15–17):**						
18	0.555	0.215–1.434	0.567	0.219–1.467	0.567	0.219–1.468
19	0.644	0.348–1.190	0.680	0.372–1.240	0.642	0.323–1.277
**Education (Ref = Some Primary):**						
Primary complete	0.400*	0.205–0.783	0.407*	0.219–0.756	0.398*	0.207–0.767
Some secondary	0.444†	0.173–1.139	0.452†	0.173–1.181	0.439†	0.171–1.125
Secondary complete	0.209**	0.077–0.573	0.214**	0.087–0.529	0.204*	0.073–0.575
**Religion (Ref = Catholic):**						
Protestant/Other	0.906	0.555–1.477	0.891	0.566–1.401	0.925	0.582–1.470
**Owns Jewelry**	0.549*	0.339–0.890	0.560*	0.347–0.902	0.564*	0.351–0.906
**Worked (Ref = No):**						
Yes	1.876**	1.356–2.596				
**Saved Regularly (Ref = No):**						
Yes	1.509	0.374–6.084				
**Work & Saving (Ref = No Work):**						
Work + No Regular Saving			1.959**	1.410–2.721		
Work + Regular Saving			1.590	1.410–2.721		
**Work Income & Saving (Ref = No Work)**						
Lower Income^a^ + No Regular Saving					1.505	0.865–2.618
Higher Income^a^ + No Regular Saving					2.640*	1.302–5.354
Work + Regular Saving					1.613	0.389–6.689
**Partner Trusts Her with Money (Ref = N**						
Yes	0.365*	0.158–0.841	0.367*	0.152–0.887	0.377*	0.149–0.953
**Husband’s Age**	1.038	0.882–1.223	1.040	0.894–1.210	1.045	0.900–1.123
**Husband Worked in Past Month: (Ref = No):**						
Yes	1.022	0.427–2.447	0.946	0.404–2.213	0.983	0.420–2.302
**Number of Children: (Ref = None):**						
1	1.587	0.503–5.009	1.587	0.522–4.824	1.635	0.531–5.037
2+	2.027	0.637–6.449	2.000	0.672–5.954	2.052	0.648–6.501

**p < .01;

*p < .05;

^†^<0.10

^a^ Lower income is defined as less than the median income of 3,001 KES (USD 35.4) and higher income is equal to or greater than this amount.

In the first model including each key independent variable separately, work was associated with 87% greater odds of violence compared to not-working (OR = 1.87, p<0<0.01), saving regularly was not associated with violence, and partner trust regarding money was associated with 63% lower odds of violence compared to not having partner trust (OR = 0.365, p<0.05). The second model included a variable that compared girls who did not work with: 1) those who worked and did not save regularly and 2) those who worked and saved regularly, controlling for the same variables. In this model, there was no significant difference between girls who worked and saved regularly and those who did not work. However, work with no regular saving was associated with 95% greater odds of violence compared to not-working (OR = 1.959, p<0.01). Lastly, a third model was estimated to examine the effect of the amount of money earned. A variable was created comparing girls who did not work with: 1) earning less than the median income (Ksh 3,001; USD 35.4) with no regular saving, 2) earning the median income or more with no regular saving, and 3) work and regular saving. Results showed that only earning a higher income with no regular saving was significantly associated with increased odds of experiencing physical violence (OR = 2.640, p<0.05). The independent protective effect of partner trust with money remained significant in both Model 2 and Model 3.

### Qualitative Results

The qualitative analysis examined respondent’s perceptions regarding the associations between women’s work and physical violence, relationship dynamics, the role of savings.

Both men and women discussed relationship dynamics that contribute to physical violence, including lack of trust and norms about power dynamics and a woman’s role in a relationship. The main reason reported for lack of trust between partners was suspected or actual infidelity. When women mistrust their partners and question them with suspicions of infidelity, this angers their male partners and triggers violence. On the other hand, men will also resort to violence when they suspect their female partners of being unfaithful.

*There are situations like not trusting in someone… if I come like 2am from searching [for money] and when you explain to her she doesn’t understand…*.*so you might disagree with her and you find that you have beaten her because she doesn’t want to listen to you*.

*Nakuru male respondent*, *age 24*, *separated*, *casual laborer*

*You might find that a man has trusted her and left her in the house; maybe she is a house wife and when the man comes*, *he find her not in the house*, *maybe she comes late*. *The man will ask her on where she went*. *So that lack of explanation will make her get beaten*. *Why*? *Because she left hoping that the husband will come late as he does but she has come early*.

*Kariobangi male respondent*, *age 25*, *never married*, *casual laborer*

Respondents also discussed the role of money in relationships and its association with IPV. Poverty, unemployment and lack of money lead to disagreements and elevated stress levels, which makes the male partners more volatile, particularly when combined with alcohol use. Some partners become violent when a woman asks a man for money, yet they do not have any. When females earn or have their own money, males may be suspicious of the source of the funds, which can lead to violence. In addition, male partners may beat their wives or girlfriends if they feel they are mismanaging or misusing money that they have given them for specific purposes, such as buying food. Male respondents were more likely to report financial issues as triggers of violence than female respondents.

*It was a slap*, *he was hungry and I went to him to ask him for money yet he didn’t have*. *I loved sweet things; I wanted him to give me 75 shillings I buy a quarter [kilo] of meat*. *Imagine he slapped me and the following day I went into labor*. *I didn’t eat*. *I gave him food*, *he ate and then we slept*.

*Kisumu female respondent*, *age 19*, *married*, *food vendor*

*Maybe you had warned her like [about] not cooking for the kids where they stay hungry and you had left her with the money and when you ask her*, *she tells you that there is no money*. *With that you have to punch her*.

*Kisumu male respondent*, *age 23*, *married*, *casual laborer*

*Like you find a man has gone to work and he has come empty handed and when he is being asked about something*, *you find that he doesn’t have…there is also stress*. *So he looks for small mistakes which force him to react*.

*Kariobangi male respondent*, *age 26*, *never married*, *teacher*

Both males and females discussed gender roles, in which men are expected to be the heads of the households and to have more power in relationships than women. When only the male works, his role is to provide for the household and the woman’s role is to ensure all household responsibilities are fulfilled and children are taken care of. When both are working, women have more power in the relationship but they are still expected to acknowledge and respect their male partners as being “above” them. Some mentioned that independent women may opt to be with a partner who is not working so as to maintain their independence. In general, women are expected to be subservient to men by being obedient, showing them respect, and accepting their responses and decisions. Many indicated that it was justifiable or necessary for a man to beat a woman when she undermines her partner or does not fulfill her wifely duties.

*If we talk about the wife being beaten by the husband*, *you may find that in the house she is not clean*, *the way the husband left his clothes on the chair they are still there*, *you have not washed for him*, *maybe you have not cooked for the children…he has come to the house you are not there*, *he went to work he has come back in the house before you yet you has not gone to work*, *that’s why many wives are beaten…*.

*Kariobangi female respondent*, *age 24*, *married and casual laborer*

*They should be obedient*, *when they are told “do this” they should do it and even if the man tells her bad things she should not be so angered*. *She should keep calm for her anger to cool down then they talk*. *They will live in peace…*. *Just being obedient because most of them are not obedient*. *She has to be slapped for her to reason well*.

*Nakuru female respondent*, *age 18*, *married*, *housewife*

*It might affect because she will feel that she is above him*. *Maybe that boy doesn’t have money and she is the one supporting him*. *She will feel that since she is the one with money she can do anything*. *So she will want to be above him*, *but you know there is no day that a woman will be above a man*. *Even if she has the money he will obviously be the head*.

*Kisumu female respondent*, *age 23*, *married*, *unemployed*

Respondents did not directly compare risk of IPV for working women versus women who were not working independent of the factors described above. However, when discussing ways to prevent IPV and benefits of financial independence and savings, they mentioned that women with access to their own money had greater choice when selecting a partner and greater ability to leave an abusive partner. Some also mentioned that working keeps women busy and reduces marital tensions. Women who work can use their own money to cater for needs that arise rather than needing to ask their partners for money, which sometimes triggers violence.

*What I think can be done…is to keep yourself busy doing something…such that these issues are eradicated*. *You know if you keep yourself busy*, *when you are from work you don’t have time to ask your husband if there is this or that*. *You know there are some when you ask for something that’s where quarrels arise*, *and beating comes*.

*Kariobangi female respondent*, *age 24*, *married*, *self employed*

*You see*, *when a girl gets a job*, *and I happen also to go to my job and I come at midnight*, *and maybe she gets back at 6pm*, *she will give me a call and tell her that I will come late so work it out with the baby I will come a bit late*. *She will understand because she knows that you have gone for work*. *She will not look around asking whether she will sleep hungry or go to borrow with such shame and such kind of things*. *If she has her job*, *she will know on how to sort herself without depending on me on many things*. *Hers is just to know that she has a man in the house and they understand each other*.

*Nakuru male respondent*, *age 24*, *married*, *casual worker*

*Let us say that if she is your girlfriend or wife*, *you cannot beat her because she already has money*. *She will ask you “why you are beating me and there is nothing you are helping me with*. *I can survive alone”*. *So that is what will make the relationship not to last*. *It will last for few minutes depending on the money she has*.

*Kariobangi male respondent*, *age 25*, *never married casual laborer*

## Discussion

Findings show that while economic empowerment in the form of work for married adolescent girls can be associated with increased risk of experiencing violence, having savings can be protective. Adjusting for other factors, results show a significant association between work and experience of physical violence, particularly working without saving regularly. Qualitative findings support quantitative results in regards to the importance of saving and having a partner who trusts his wife with money, both of which were associated with reduced odds of experiencing IPV. They also elucidate some of the contextual factors associated with IPV as described in the ecological model, the role of power dynamics in relationships, gender norms, couple conflict, poverty, alcohol abuse.

Findings are consistent with the program intervention targeting girls in the study sites based on the asset-building theory to economically empower girls through financial education and savings while also building their health and social assets. A similar program in Uganda showed that this multi-sectoral approach reduced girls’ risk of experiencing sexual harassment as compared to providing economic strengthening alone [13]. Through safe-spaces groups, girls built their social networks and were connected with a mentor from their community who trained them on financial education and reproductive health, including issues of gender, preventing gender-based violence and relationships. A randomized-controlled trial in Cote d’Ivoire also showed that addressing household gender inequities alongside economic empowerment programming has the potential to reduce IPV among women [12].

The role of savings for vulnerable married girls, especially in the context of broader economic strengthening programming, may play a key role in risk mitigation. The association between regular savings and protection from IPV is important for the messaging that is included on financial education for girls to ensure that they save regularly and frequently. One consistent finding in the quantitative and qualitative studies was that having a partner that trusts the girl with money is a protective factor. Therefore, it is also important to include messaging related to communication and management of conflicts regarding money within relationships. In addition, the type of financial services that are offered to girls could potentially impact regular saving, for example, contract savings in which a certain amount is required each month. Further exploration of the differences between formal and informal savings in relation to risk of IPV would be a useful contribution to understanding this association.

Findings indicate that given the gender norms in this patriarchal society, and financial difficulties faced by families living in informal settlements, financial conflicts can be triggers of violence in marriages. However, women’s management of and access to financial resources can potentially help to reduce this risk. Further research is needed to examine whether there is a causal relationship between working and experience of IPV for married adolescent girls residing in slums in Kenya. At the end of the two-year intervention period, girls included in the quantitative study will be interviewed again at endline and additional analysis will be conducted to examine the effect of employment on the incidence of IPV, and the impact of the savings component of the intervention.

The main limitation of the quantitative study is the cross-sectional data, which makes it difficult to determine the timing of events and to infer causality. Using this data it is not possible to determine whether girls engaged in work activities before they experienced physical violence in the previous six months. Self-report data is subject to recall bias and the responses may be influenced by the way the questions are posed. Although the multivariate analysis controls for demographic characteristics, it does not account for unobserved heterogeneity and other background factors that might make girls who work systematically different than girls who do not work. The qualitative study would have been stronger if the in-depth interviews would have included more specific questions on the association between work and violence and the role of trust. Nonetheless, the results provided useful information to aid in understanding the quantitative results.

## Supporting Information

S1 File Economic Assets and GBV Baseline Survey. (PDF)

S2 File In-Depth Interview Guide for Adolescent Girls. (PDF)

S3 File In-Depth Interview Guide for Young Men. (PDF)
