# Correction: Is Working Risky or Protective for Married Adolescent Girls in Urban Slums in Kenya? Understanding the Association between Working Status, Savings and Intimate-Partner Violence

**DOI:** 10.1371/journal.pone.0158250

**Published:** 2016-06-22

**Authors:** Eunice Muthengi, Tabither Gitau, Karen Austrian

There is an error in Table 4. The value listed for “Work + Regular Saving” under “Model 2” should be “0.382–6.623”. Please see the corrected Table 4 here.

**Table 4:** Multivariate Logistic Regression Results Predicting Experience of Physical Violence in the Previous Six Months, Married Girls, Age 15–19 (N = 445).

	Model 1: Original Model		Model 2: Work Status and Saving Status Interaction		Model 3: High/Low Work Income and Saving Interaction	
	OR	[95% CI]	OR	[95% CI]	OR	[95% CI]
**Age (Ref = 15–17):**						
18	0.555	0.215–1.434	0.567	0.219–1.467	0.567	0.219–1.468
19	0.644	0.348–1.190	0.680	0.372–1.240	0.642	0.323–1.277
**Education (Ref = Some Primary):**						
Primary complete	0.400*	0.205–0.783	0.407*	0.219–0.756	0.398*	0.207–0.767
Some secondary	0.444†	0.173–1.139	0.452†	0.173–1.181	0.439†	0.171–1.125
Secondary complete	0.209**	0.077–0.573	0.214**	0.087–0.529	0.204*	0.073–0.575
**Religion (Ref = Catholic):**						
Protestant/Other	0.906	0.555–1.477	0.891	0.566–1.401	0.925	0.582–1.470
**Owns Jewelry**	0.549*	0.339–0.890	0.560*	0.347–0.902	0.564*	0.351–0.906
**Worked (Ref = No):**						
Yes	1.876**	1.356–2.596				
**Saved Regularly (Ref = No):**						
Yes	1.509	0.374–6.084				
**Work & Saving (Ref = No Work):**						
Work + No Regular Saving			1.959**	1.410–2.721		
Work + Regular Saving			1.590	0.382–6.623		
**Work Income & Saving (Ref = No Work)**						
Lower Income^a^ + No Regular Saving					1.505	0.865–2.618
Higher Income^a^ + No Regular Saving					2.640*	1.302–5.354
Work + Regular Saving					1.613	0.389–6.689
**Partner Trusts Her with Money (Ref = N**						
Yes	0.365*	0.158–0.841	0.367*	0.152–0.887	0.377*	0.149–0.953
**Husband’s Age**	1.038	0.882–1.223	1.040	0.894–1.210	1.045	0.900–1.123
**Husband Worked in Past Month: (Ref = No):**						
Yes	1.022	0.427–2.447	0.946	0.404–2.213	0.983	0.420–2.302
**Number of Children: (Ref = None):**						
1	1.587	0.503–5.009	1.587	0.522–4.824	1.635	0.531–5.037
2+	2.027	0.637–6.449	2.000	0.672–5.954	2.052	0.648–6.501

**p < .01;

*p < .05;

^†^<0.10

^a^ Lower income is defined as less than the median income of 3,001 KES (USD 35.4) and higher income is equal to or greater than this amount.
