# Supplementary material for: Is Working Risky or Protective for Married Adolescent Girls in Urban Slums in Kenya? Understanding the Association between Working Status, Savings and Intimate-Partner Violence
Source: PLoS One. 2016 May 27;11(5):e0155988. doi: 10.1371/journal.pone.0155988 (PMC4883769; doi:10.1371/journal.pone.0155988)
Supplement: S1 File — (PDF) [file pone.0155988.s001.pdf]

Subject ID Number _____________

BASELINE STUDY
Adolescent Girls in Kenya

IDENTIFICATION	CODES
**Research Site:** Intervention = 1<br>Comparison = 2	
**Study Site:** Kisumu = 1<br>Thika = 2<br>Nakuru = 3<br>Nairobi= 4	
**Location of Interview:** ____	
**DATE (day/month/year):** |_____|_____|_____|	
**INTERVIEWER NAME:** ____	
**INTERVIEWER CODE:** ____	
**RECORD TIME INTERVIEW BEGINS:**<br>Hour: |____|____| Minutes: |____|____|	

VISIT OUTCOME	CODES	LANGUAGE USED IN INTERVIEW	CODES
1= COMPLETED<br>2= LANGUAGE INCOMPATIBLE<br>3= PARTIALY COMPLETED<br>4= POSTPONED<br>5=REFUSED<br>96=OTHER (SPECIFY)<br>_______________________		1 = ENGLISH<br>2 = KISWAHILI<br>3 = BOTH ENGLISH & KISWAHILI<br><br>96=OTHER (SPECIFY)<br>_________________	

SUPERVISOR SIGNATURE AND DATE_________________________________	______________
DATA EDITOR SIGNATURE AND DATE_________________________________	______________
DATA ENTRY SIGNATURE AND DATE__________________________________	______________

Subject ID Number _____________

100. Basic Demographic and Household Information

101. In what month and year were you born? / *Ulizaliwa mwezi na mwaka gani?*

MONTH___________ YEAR_____________

102. How old are you? / *Je una miaka mingapi?* (**COMPARE AND CORRECT 101 AND/OR 102 IF INCONSISTENT**)

AGE IN COMPLETED YEARS___________________ ***IF LESS THAN 15 END INTERVIEW***

103. What is your religion? / *Dini yako ni gani?*

1=CATHOLIC
2=PROTESTANT/ OTHER CHRISTIAN
3=TRADITIONAL
4=MUSLIM
5=NO RELIGION
96=OTHER (SPECIFY) ____

104. What village do you live in? / *Unaishi kijiji gani?*

VILLAGE NAME___________________

105. Are you currently married? / *Je, kwa sasa umeolewa?*

1=NO
2=YES **(GO TO Q108)**

106. Have you ever been married? / *Je, umewahi kuolewa mbeleni?*

1=NO
2=YES **(GO TO Q108)**

107. Have you ever lived with a boyfriend? / *Je, umewahi kuishi na mpenzi wa kiume?*

1=NO **(GO TO Q110)**
2=YES **(GO TO Q109)**

108. How old were you when you got married? / *Je, uliolewa ukiwa na miaka mingapi?*

AGE IN YEARS _______________________________________ **(GO TO Q110)**

109. How old were you when you started living with your boyfriend? / *Je,ulikuwa na miaka mingapi ulipoanza kuishi na mpenzi wako?*

AGE IN YEARS _______________________________________

110. Is your mother alive? / *Je, mama yako yungali hai?*
1=NO
2=YES **(GO TO Q112)**
88=DON'T KNOW **(GO TO Q112)**

111. How old were you when your mother died? *Je, ulikuwa na umri gani mama yako alipofariki?*

AGE IN YEARS _____________

8888=DON'T KNOW

112. Is your father alive? / *Je, baba yako yungali hai?*

1=NO
2=YES **(GO TO Q114)**
88=DON'T KNOW **(GO TO Q114)**

113. How old were you when your father died? *Je, ulikuwa na umri gani baba yako alipofariki?*

AGE IN YEARS _____________
8888=DON'T KNOW

114. Have you ever attended school? / *Je, umewahi kwenda shule?*
(INCLUDING NON-FORMAL SCHOOL)

1=NO (**GO TO Q120)**
2=YES

115. Are you currently in school? / *Je, kwa sasa uko shuleni?*

1=NO **(GO TO Q119)**
2=YES

116. What class/form are you in? / *Je uko darasa/kidato cha ngapi?*

1=CLASS 1	10=FORM 2
2=CLASS 2	11=FORM 3
3=CLASS 3	12=FORM 4
4=CLASS 4	13=PRE-SECONDARY VOCATIONAL COURSE
5=CLASS 5	14=POST-SECONDARY VOCATIONAL COURSE
6=CLASS 6	15=COLLEGE
7=CLASS 7	16=UNIVERSITY
8=CLASS 8	17=NON-FORMAL SCHOOL
9=FORM 1	96=OTHER (Specify) ______________________________

117. Have you ever had a break in your schooling for six months or more? / *Je, umewahi kukatiza masomo yako kwa muda wa miezi sita au zaidi?*

1=NO **(GO TO Q121)**
2=YES

118. What is the **MAIN** reason that there was a break in your schooling? / *Je, ni nini kilisababisha ukatize masomo yako?*

0=FAMILY COULD NOT AFFORD – *Jamii yangu haingeweza kunisomesha*
1=GOT MARRIED - *Niliolewa*
2=TOO MANY DOMESTIC RESPONSIBILITIES – *Nilikuwa na kazi nyingi za nyumbani*
3=SCHOOL TOO FAR / NO SCHOOL IN VICINITY – *Shule iko mbali*
4=FAMILY DOES NOT APPROVE/SEE BENEFIT – *Jamii yangu haioni umuhimu wa kusoma*
5=NO SCHOOL PLACES AVAILABLE – *Hakuna nafasi shuleni*
6=GOT PREGNANT – *Nilipata mimba*
7=ILLNESS - Ugonjwa
96=OTHER (SPECIFY) ____
88=DON'T KNOW - *Sijui*

(GO TO Q121)

119. What is the highest level of education that you reached? / *Je, umesoma hadi kiwango gani?*

1=CLASS 1	10=FORM 2
2=CLASS 2	11=FORM 3
3=CLASS 3	12=FORM 4
4=CLASS 4	13=PRE-SECONDARY VOCATIONAL COURSE
5=CLASS 5	14=POST-SECONDARY VOCATIONAL COURSE
6=CLASS 6	15=COLLEGE
7=CLASS 7	16=UNIVERSITY
8=CLASS 8	17=NON-FORMAL SCHOOL
9=FORM 1	96=OTHER (Specify) _____________________________

120. What is the **MAIN** reason that you are not in school? / *Je, ni sababu gani kuu imekufanya usiende shule?*

0=FAMILY COULD NOT AFFORD – *Jamii yangu haingeweza kunisomesha*
1=GOT MARRIED - *Niliolewa*
2=TOO MANY DOMESTIC RESPONSIBILITIES – *Nilikuwa na kazi nyingi za nyumbani*
3=SCHOOL TOO FAR / NO SCHOOL IN VICINITY – *Shule iko mbali*
4=FAMILY DOES NOT APPROVE/SEE BENEFIT – *Jamii yangu haioni umuhimu wa kusoma*
5=NO SCHOOL PLACES AVAILABLE – *Hakuna nafasi shuleni*
6=GOT PREGNANT – *Nilipata mimba*
7=FINISHED SCHOOL – *Nimemaliza shule*
96=OTHER (SPECIFY) ____
88=DON'T KNOW - *Sijui*

121. Can you read and understand a letter or newspaper easily, with difficulty, or not at all? / *Je, unaweza kusoma barua ama gazeti na kuelewa kwa urahisi, kwa shida, au hauwezi kabisa?*

1=EASILY – *Kwa urahisi*
2=WITH DIFFICULTY – *Kwa shida*
3=NOT AT ALL – *Siwezi kamwe*

122. Now I want to ask you a few questions about the other people who live in your house. For each person that lives with you I will ask about their schooling and employment. Other than you, how many people live in your household?/ *Sasa, ningependa kukuuliza maswali machache kuhusu watu wengine mnaoishi nao. Nitauliza kuhusu elimu na kazi ya kila mtu ambaye unaishi naye. Isipokuwa wewe, ni watu wangapi wanaoishi kwa nyumbe yenu?*

NUMBER OF PEOPLE **(EXCLUDING RESPONDENT) __________(IF NONE=0 GO TO Q201)**

(START BY ASKING WHO THEY LIVE WITH, CONTINUE TO PROBE ABOUT OTHERS WHO LIVE IN THE HOUSEHOLD UNTIL ALL WHO LIVE WITH THE RESPONDENT HAVE BEEN IDENTIFIED).

(HOUSEHOLD MEMBER IS DEFINED AS SOMEONE WHO HAS SPENT THE MAJORITY OF THE NIGHTS IN THE PAST MONTH SLEEPING IN THAT HOUSE)

(ASK SCHOOL/WORK QUESTIONS – 122e – 122f – ONLY OF PERSONS AGE 5 AND ABOVE).

**122a**	**122b**	**122c**	**122d**	**122e**	**122f**
Name / *Jina*	Age / *Umri*	Sex / *Jinsia*<br><br>F = 1<br>M = 2	Relationship to Respondent / *Uhusiano* (see codes)	Highest Level Education Reached / *Kiwango cha elimu cha juu*	Have they worked for pay in the last month? / *Je, wamewahi kufanya kazi ya kulipwa mwezi uliopita?*

				alichofikia (see codes)			
					**No**	**Yes**	**Don't Know**
					1	2	88
					1	2	88
					1	2	88
					1	2	88
					1	2	88
					1	2	88
					1	2	88
					1	2	88
					1	2	88

CODES 122d:

0=MOTHER
1=FATHER
2=BROTHER
3=SISTER
4=GRANDMOTHER
5=GRANDFATHER
6=COUSIN
7=AUNT
8=UNCLE
9=FRIEND
10=BOYFRIEND/HUSBAND
11=OWN CHILD
12=EMPLOYER
13=GUARDIAN
96=OTHER (SPECIFY)_______________________

CODES 122e:

1=NO EDUCATION
2=NURSERY SCHOOL
3=PRIMARY SCHOOL
4=SECONDARY SCHOOL
5=VOCATIONAL/TECHNICAL TRAINING
6=UNIVERSITY
7=NON-FORMAL SCHOOL
88=DON'T KNOW

200. HOUSEHOLD ASSETS

201. What is the main material used for the EXTERIOR WALLS of the house you live in? / *Je, KUTA za nyumba unayoishi zimejengwa na vifaa gani?*

1=MUD - *Matope*
2=CEMENT - *Simiti*
3=CORRUGATED IRON – *Mabati*
4=WOOD - *Mbao*
96=OTHER (SPECIFY) ___________________________

202. What is the main material used for the ROOF of the house you live in? / *Je, PAA ya nyumba unayoishi imejengwa na vifaa gani?*

1=CORRUGATED IRON - *Mabati*
2=PLASTIC - *Plastiki*

3=PLYWOOD – *Mbao*
4=TILES - *Vigae*
96=OTHER (SPECIFY) ___________________________

203. What is the main material used for the FLOOR of the house you live in? / *Je, SAKAFU ya nyumba unayoishi imejengwa na nini?*

1=MUD - *Matope*
2=CEMENT - *Simiti*
3=WOOD/CEMENT WITH ADDITIONAL COVERING – *Mbao / simiti na imefunikwa*
96=OTHER (SPECIFY) __________________________________

204. How many rooms in your household are used for sleeping? / *Je, kuna vyumba vingapi vinavyo tumika kwa kulala katika nyumba yenu?*

NUMBER OF ROOMS ________________________

205. How many people share the same sleeping room with you? / *Je, mnalala watu wangapi katika chumba unacholala?*

NUMBER OF PEOPLE ________________________

Now I want to ask you about different items that you may or may not have in your household, and who owns them. / *Sasa, nataka nikuulize kuhusu vitu ambavyo mnaweza kuwa navyo au msiwenavyo katika nyumba yenu, na nivya nani*.

	206. Does anyone in the **household** that you live in own or have these items? / *Je, watu mnaoishi nao wana vitu hivi?* **(IF NO ON Q206, GO TO NEXT ITEM, SKIP Q207, Q208)**	207. Do you **personally own** or have these items? / *Je, wewe binafsi una vitu hivi?* **(IF NO, GO TO NEXT ITEM, SKIP Q208)**	208. If you need to, can you sell this item without anyone else's permission? / *Je, unaweza kuviuza bila kuomba ruhusa?* 1=NO 2=YES	
A. ELECTRICITY / *Stima*	1=NO 2=YES			
B. RADIO / *Radio*	1=NO 2=YES	1=NO 2=YES	1	2
C. TELEVISION / *runinga/TV*	1=NO 2=YES	1=NO 2=YES	1	2
D. TELEPHONE/MOBILE PHONE / *Simu*	1=NO 2=YES	1=NO 2=YES	1	2
E. KEROSENE LAMP / *Taa ya mafuta*	1=NO 2=YES	1=NO 2=YES	1	2
F. BED / *Kitanda*	1=NO 2=YES	1=NO 2=YES	1	2
G. BICYCLE / *Baiskeli*	1=NO 2=YES	1=NO 2=YES	1	2
H. MOTORCYLE / *Pikipiki*	1=NO 2=YES	1=NO 2=YES	1	2
I. CAR / *Gari*	1=NO 2=YES	1=NO 2=YES	1	2
J. THE HOUSE YOU LIVE IN /	1=NO 2=YES	1=NO 2=YES	1	2

	206. Does anyone in the **household** that you live in own or have these items? / *Je, watu mnaoishi nao wana vitu hivi?* **(IF NO ON Q206, GO TO NEXT ITEM, SKIP Q207, Q208)**	207. Do you **personally own** or have these items? / *Je, wewe binafsi una vitu hivi?* **(IF NO, GO TO NEXT ITEM, SKIP Q208)**	208. If you need to, can you sell this item without anyone else's permission? / *Je, unaweza kuviuza bila kuomba ruhusa?* 1=NO 2=YES	
Nyumba unayoishi				
K. LAND / *Shamba*	1=NO 2=YES	1=NO 2=YES	1	2
L. SMALL LIVESTOCK / *Mifugo ndogo (i.e. poultry, goats / kuku, mbuzi)*	1=NO 2=YES	1=NO 2=YES	1	2
M. LARGE LIVESTOCK / *Mifugo kubwa (i.e. cows, pigs / ng'ombe, nguruwe)*	1=NO 2=YES	1=NO 2=YES	1	2
N. REFRIGERATOR / *Friji*	1=NO 2=YES	1=NO 2=YES	1	2
O. TOOLS/MACHINE PEOPLE USE TO EARN MONEY / *Mitambo ya kuleta pesa*	1=NO 2=YES	1=NO 2=YES	1	2
P. JEWELRY / *Pete, ushaanga*	1=NO 2=YES	1=NO 2=YES	1	2

209. What is the main source of drinking water for members of your household? / *Je, maji mnayokunywa nyumbani kwenu inatoka wapi?*

1=PIPED WATER PIPED INTO RESIDENCE/COMPOUND/PLOT – *Maji hadi kwa ploti*
2=PUBLIC TAP WHERE WATER IS FREE – *Mfereji wa maji ya bure*
3=PUBLIC TAP WHERE WATER IS BOUGHT – *Maji ya mfereji yanayouzwa*
4=WELL WATER IS BOUGHT – *Maji ya kisima yanayouzwa*
5=WELL ON RESIDENCE/PLOT – *Kisima kwenye ploti*
6=PUBLIC WELL – *Kisima cha kila mtu*
7=SURFACE WATER/RIVER/STREAM/POND/LAKE – *Mto / Kijito / Ziwa la maji*
8=RAINWATER – *Maji ya mvua*
96=OTHER (SPECIFY) _________________________________

210. Do you have an ID with your photo on it? */ Je, una kipande kilicho na picha yako?*

1=NO
2=YES

300. Working & Savings Activities

Now I am going to ask you several questions about different issues related to your use of money, including making money, spending money, and savings. We know that some girls have money to spend and save and others don't. So, there are no right or wrong answers, just answer as honestly as you can. / *Sasa, nitakuuliza maswali kuhusu unavyotumia pesa zako, pamoja na kujipatia pesa, kutumia na*

kuwekeza akiba. Tunajua kwamba baadhi ya wasichana wana pesa na kutumia na kuwekeza na wengine hawana. Kwa hivyo, hakuna jibu lililosahihi ama kosa, jibu tu jinsi unavyoelewa na kwa uaminifu.
301. Where do you get the money that you spend? / *Je, unapata wapi pesa unazotumia?* **(READ ALL ANSWERS ALOUD AND CIRCLE ALL THAT APPLY)**

1=MOTHER - *Mama*
2=FATHER - *Baba*
3=GUARDIAN YOU LIVE WITH – *Mlezi unayeishi naye*
4=OTHER RELATIVES – *Watu wa jamii*
5=FRIEND - *Rafiki*
6=BOYFRIEND – *Mpenzi wa kiume*
7=HUSBAND – *Mume wako*
8=SUGAR DADDY – *Mzee mpenzi*
9=STRETCHING MONEY (I.E. KEEPING CHANGE FROM WHEN SENT TO KIOSK) – *Kutorudisha pesa baada ya kununua kitu*
10=OWN SAVINGS – *Akiba ya kibinafsi*
11=CHAMA - *Chama*
12=CASUAL JOBS (I.E. WASHING CLOTHES, PLAITING HAIR) – *Kibarua (kama kufua nguo au kusonga nywele)*
13=STEADY JOB – *Kazi ya kuajiriwa*
14= OTHER NON-RELATIVE
15= DON'T HAVE/SPEND MONEY – *Sina ama situmii pesa*

In the past month, did you spend money on this item? */ Katika MWEZI mmoja uliopita, ulitumia pesa kununua hii kitu?*

(READ EACH ITEM ALOUD, ASK YES OR NO. ***NOTE****: Clarify if it is a personal spending need or for someone else if it is not clear from type of expenditure)*

(IF NO ON Q302, GO TO NEXT ITEM, SKIP Q303 TO Q304.)

	**Q302.** In the past month, did you spend money on this item? Katika MWEZI mmoja uliopita, ulitumia pesa kununua hii kitu?	**Q303.** About how much do you spend in one month on this item?/ *Unatumia takriban kiasi gani cha pesa katika mwezi mmoja?*	**Q304.** Do you decide to buy this item on your own, together with someone , or someone else decides for you? *Wewe mwenyewe hujiamulia kununua kitu hiki, huamua pamoja na mtu mwengine au mtu mwengine hukuamulia?*
	NO = 1<br>YES = 2	KSH	1= YOU ALONE<br>2 = YOU TOGETHER WITH SOMEONE<br>3 = SOMEONE ELSE
a. FOOD, CHIPS, SWEETS, DRINKS/SODA FOR YOURSELF / *CHAKULA, CHIPS, PIPI, VINYWAJI/SODA ZAKO*			
b. FOOD, CHIPS, SWEETS, DRINKS/SODA FOR SOMEONE ELSE / *CHAKULA, CHIPS, PIPI,*			

VINYWAJI/SODA ZA MTU MWENGINE			
c. CLOTHES/SHOES FOR YOURSELF/ *NGUO/VIATU ZAKO*			
d. CLOTHES/SHOES FOR SOMEONE ELSE/ *NGUO/VIATU ZA MTU MWENGINE*			
e. PERSONAL ITEM (BEAUTY PRODUCTS, SANITARY TOWELS, UNDERWEAR)/ VITU VYA BINAFSI *(BIDHAA ZA UREMBO, TAULO YA HEDHI, CHUPI)*			
f. BEAUTY SERVICES/ *HUDUMA YA UREMBO*			
g. CREDIT/AIRTIME/ *MUDA WA MAONGEZI*			
h. ENTERTAINMENT/ *BURUDANI*			
i. TRANSPORT/ *USAFIRI*			
j. RENT/ *KODI*			

In the past YEAR, did you spend money on this item? */ Katika MWAKA mmoja uliopita, ulitumia pesa kununua hii kitu?*

(READ EACH ITEM ALOUD, ASK YES OR NO. ***NOTE****: Clarify if it is a personal spending need or for someone else if it is not clear from type of expenditure)*

(IF NO ON Q305, GO TO NEXT ITEM, SKIP Q306 TO Q307.)

	**Q305.** In the past year, did you spend money on this item? Katika MWAKA mmoja uliopita, ulitumia pesa kununua kitu hiki?	**Q306.** About how much do you spend in one YEAR on this item / Unatumia takriban kiasi gani cha pesa kwa hiki kitu katika mwaka mmoja?	**Q307.** Do you decide to spend this money on your own, together with someone, or someone else decides for you? *Wewe mwenyewe hujiamulia kutumia hizi pesa, huamua pamoja na mtu mwengine au mtu mwengine hukuamulia?*
	NO = 1<br>YES = 2	KSH	1= YOU ALONE<br>2 = YOU TOGETHER WITH SOMEONE<br>3 = SOMEONE ELSE
a. SCHOOL SUPPLIES/ *VIFAA VYA SHULE*			
b. SCHOOL FEES FOR YOURSELF/ *ADA*			

YAKO YA SHULE/KARO YAKO YA SHULE			
c. SCHOOL FEES FOR SOMEONE ELSE/ *ADA YA MTU MWENGINE YA SHULE/KARO YA MTU MWENGINE YA SHULE*			
d. EXAM FEES/ *ADA YA MTIHANI*			
e. INVEST IN BUSINESS/ *KUWEKEZA KATIKA BIASHARA*			
f. HEALTH FOR YOURSELF (MEDICAL SERVICES, MEDICINE, FAMILY PLANNING, HOSPITAL)/ *AFYA YAKO (HUDUMA YA MATIBABU, TIBA, KUPANGA UZAZI, HOSPITALI)*			
g. HEALTH FOR SOMEONE ELSE (MEDICAL SERVICES, MEDICINE, FAMILY PLANNING, HOSPITAL)/ *AFYA YA MTU MWENGINE (HUDUMA YA MATIBABU, TIBA, KUPANGA UZAZI HOSPITALI)*			
h. FUNERAL/ *MAZISHI*			
i. MOBILE PHONE/ *RUNUNU/SIMU YA MKONO*			

308. Some people do different kind of work that they get paid for either with money, goods, or services. This can be regular work or irregular work. You can work for yourself or for someone else. Have you ever done any work for which you were paid in cash or with goods or services? / *Watu wengine hufanya kazi tofauti ambapo hulipwa pesa, bidhaa, au huduma. Inaweza kuwa kazi ya kila siku au kibarua. Unaweza kujiajiri ama kuajiriwa na mtu mwingine. Je, umewahi kufanya kazi na ukalipwa pesa, ama bidhaa ama huduma zingine?*

1=NO **(GO TO Q315)**
2=YES

309. How old were you when you started this work? / *Je, ulikuwa na miaka mingapi ulipoanza kazi hii?*

AGE IN YEARS ___________________________

310. Who decides how the money that you earn will be spent? / *Ni nani ambaye hutoa uamuzi kuhusu jinsi ambavyo pesa unazopata zitakavyotumiwa: Ni wewe binafsi, wewe pamoja na mtu mwengine, au mtu mwengine?*

1=YOU ALONE
2=YOU WITH SOMEONE ELSE
3=SOMEONE ELSE

Please tel me about the different kinds of activities or work you have done in the past one year: / *Tafadhali orodhesha shughuli zote tofauti tofauti au kazi uliyofanya katika mwaka mmoja uliopita.*

(FOR EACH INCOME SOURCE, ASK Q312 TO Q314)

**Q. 311A.** Income Source / *Asili ya pato*		**Q. 312A.** When you have "income source" about how much did you earn in one month from this source? / *Ukiwa na asili ya ,pato, je, ni takriban kiasi gani cha pesa ulizozipata katika mwezi mmoja kutokana na asili/njia hiyo?*	**Q. 313A.** In how many months from the past one year did you receive this income? / *Ni katika miezi mingapi uliyopokea asili ya pato hili katika mwaka mmoja uliopita?*	**Q. 314A.** Were you hired as staff, was it a temporary job (kibarua) or were you running your own business? / Ulikuwa umeandikwa kazi, ama ulikuwa ukifanya kibarua au ulikuwa ukifanya biashara yako ya kibinafsi?
		KSH	MONTHS	1=HIRED STAFF<br>2=TEMPORARY JOB (KIBARUA)<br>3= RUNNING OWN BUSINESS
a. Domestic work (Maid)/ Mfanya kazi wa nyumbani	1=NO<br>2=YES			
b. Wash clothes only/ Kufua nguo pekee	1=NO<br>2=YES			
c. Wash utensils only/ Kuosha vyombo pekee	1=NO<br>2=YES			
d. Wash house only/ Kuosha nyumba pekee	1=NO<br>2=YES			
e. Fetching water/ Kuchota maji	1=NO<br>2=YES			
**Q. 311B.** Income Source /		**Q. 312B.** When you have "income source"	**Q. 313B.** In how many months from the	**Q. 314B.** Were you hired as staff, was it a temporary job

Asili ya pato		about how much did you earn in one month from this source? / *Ukiwa na asili ya ,pato, je, ni takriban kiasi gani cha pesa ulizozipata katika mwezi mmoja kutokana na asili/njia hiyo?*	past one year did you receive this income? / *Ni katika miezi mingapi uliyopokea asili ya pato hili katika mwaka mmoja uliopita?*	(kibarua) or were you running your own business? / Ulikuwa umeandikwa kazi, ama ulikuwa ukifanya kibarua au ulikuwa ukifanya biashara yako ya kibinafsi?
		KSH	MONTHS	1=HIRED STAFF<br>2=TEMPORARY JOB (KIBARUA)<br>3= RUNNING OWN BUSINESS
f. Plait hair/beauty/ Kusuka nywele/urembo	1=NO<br>2=YES			
g. Babysit/child care/ Kuchunga watoto	1=NO<br>2=YES			
h. Work in a hotel/restaurant/ Kazi ya hoteli	1=NO<br>2=YES			
i. Work in a farm/ Kazi ya shamba	1=NO<br>2=YES			
j. Packaging food or drinks/ Ufungaji wa chakula na vinywaji	1=NO<br>2=YES			
**Q. 311C**<br>Income Source /<br>*Asili ya pato*		**Q. 312C.**<br>When you have "income source" about how much did you earn in one month from this source? / *Ukiwa na asili ya ,pato, je, ni takriban kiasi gani cha pesa ulizozipata katika mwezi mmoja kutokana na asili/njia hiyo?*	**Q. 313C**.<br>In how many months from the past one year did you receive this income? / *Ni katika miezi mingapi uliyopokea asili ya pato hili katika mwaka mmoja uliopita?*	**Q. 314C.**<br>Were you hired as staff, was it a temporary job (kibarua) or were you running your own business? / Ulikuwa umeandikwa kazi, ama ulikuwa ukifanya kibarua au ulikuwa ukifanya biashara yako ya kibinafsi?
		KSH	MONTHS	1=HIRED STAFF<br>2=TEMPORARY JOB (KIBARUA)<br>3= RUNNING OWN BUSINESS

k. Selling things/ Kuuza vitu	1=NO<br>2=YES			
l. Work in office/ Kazi ya ofisi	1=NO<br>2=YES			
m. Other types of own business/ Biashara zingine za kibinafsi	1=NO<br>2=YES			
n. Other types of temporary job/ Kazi ziingine za kibarua	1=NO<br>2=YES			
o. Other type of formal employment/ Kazi zingine za kuandikwa	1=NO<br>2=YES			

315. About how much money do you currently have put aside? / *Je, ni pesa kiasi gani ambacho umetenga kando?*

_______________ KSh
0=NONE **(GO TO Q317)**

316. What are you saving for? / *Je, unajiwekea akiba ili ufanyie nini pesa hizo?* **(CIRCLE ALL THAT APPLY. PROBE – 'ANYTHING ELSE?')**

1=EMERGENCIES – *Kwa ajili ya dharura*
2=PERSONAL ITEMS (CLOTHES, PADS) – *Kujinunulia bidhaa*
3=HOUSEHOLD EXPENSES (RENT, FOOD) – *Kununua vitu vya nyumbani*
4=EDUCATION - *Kujisomesha*
5=OWN BUSINESS – Biashara yangu binafsi
6=FAMILY BUSINESS – *Biashara ya jamii*
96=OTHER (SPECIFY) __________________________________

317. In the past six months have you saved any money? / *Je, umewahi kuweka akiba ya pesa zako kwa miezi sita iliyopita?*

1=NO **(GO TO Q400)**
2=YES

318. Would you say that you saved on a **weekly basis** always, usually, sometimes, or never? / *Je unaweza kusema kwamba unajiwekea akiba* ***kila wiki*** *kila wakati, kwa kawaida, wakati mwingine, ama kamwe hujawahi?*

1=ALWAYS – *Kila wakati*
2=USUALLY – *Kwa kawaida*
3=SOMETIMES – *Wakati mwingine*
4=NEVER – *Kamwe sijawahi* **(GO TO Q320)**

319. On average, about how much money do you save per week? / *Je, unajiwekea akiba ya pesa ngapi hivi kila wiki?*

_______________ KSh

320. In the past six months, in which of the following places have you saved your money? */ Katika miezi sita iliyopita, umeweka akiba yako pahali pepi kati ya palipotajwa hapa chini?*

(READ EACH ITEM ALOUD. ASK YES OR NO)
(IF NO ON Q321, GO TO NEXT ITEM, SKIP Q322, Q323)

	**Q321A.** In the past six months, in which of the following places have you saved your money? */ Katika miezi sita iliyopita, umeweka akiba yako pahali pepi kati ya palipotajwa hapa chini?*	**Q322.** About how many shillings do you currently have saved in this place? / *Ikiwa ndiyo-Ni takriban shilingi ngapi ulizoweka kama akiba mahali hapa?*	**Q323.** In the past one year, how often did you put money in this place: once a week, once a month, a few times a year, once in the past year?/ *Ikiwa ndiyo-Katika mwaka mmoja uliopita, ni mara ngapi ulipoweka pesa mahali hapa: mara moja kwa wiki, mara moja kwa mwezi, mara chache kwa mwaka, maramoja kwa mwaka uliopita?*
		KSH	1 = Once a week<br>2 = Once a month<br>3 = A few times a year<br>4 = Once in the past year
A. HOMEBANK / *benki ya nyumbani*	1=NO<br>2=YES		
B. UNDER THE MATTRESS/HOLE IN THE GROUND/ *chini ya godoro/shimo chini ya ardhi*	1=NO<br>2=YES		
C. IN A BOX/BAG / *ndani ya sanduku/boksi/mfuko*	1=NO<br>2=YES		
D. WITH A FRIEND/ *na rafiki*	1=NO<br>2=YES		
E. WITH PARENTS/GUARDIAN / *na wazazi/mlezi*	1=NO<br>2=YES		
	**Q321B.** In the past six months, in which of the following places have you saved your money? */ Katika miezi sita iliyopita, umeweka*	**Q322.** About how many shillings do you currently have saved in this place? / *Ikiwa ndiyo-Ni*	**Q323.** In the past one year, how often did you put money in this place: once a week, once a month, a few times a year, once in the past year?/ *Ikiwa ndiyo-Katika mwaka mmoja uliopita, ni mara ngapi ulipoweka*

	akiba yako pahali pepi kati ya palipotajwa hapa chini?	*takriban shilingi ngapi ulizoweka kama akiba mahali hapa?*	*pesa mahali hapa: mara moja kwa wiki, mara moja kwa mwezi, mara chache kwa mwaka, maramoja kwa mwaka uliopita?*
		KSH	1 = Once a week<br>2 = Once a month<br>3 = A few times a year<br>4 = Once in the past year
F. SAVINGS GROUP/CHAMA / *kundi la uwekezaji akiba/chama cha uwekezaji akiba*	1=NO<br>2=YES		
G. BANK ACCOUNT /*akaunti ya benki*	1=NO<br>2=YES		
H. SHOPKEEPER / *mwenye duka*	1=NO<br>2=YES		
I. AGRICULTURAL INVESTMENTS/ *tega uchumi za kilimo*	1=NO<br>2=YES		
J. MPESA	1=NO<br>2=YES		
K. OTHER / *nyingine*	1=NO<br>2=YES		

324. Now I want you to think about the times that you've used your savings. In the past six months, about how often do you take money out of your savings to use – would you say once a week, once a month, once in every couple of months, once, or never./ *Kwa sasa ninataka ufikirie kuhusu nyakati ambazo umetumia akiba zako. Katika miezi sita iliyopita, kwa kawaida unachukua pesa kutoka kwa akiba yako ili utumie kwa takriban mara ngapi? – Ungeweza kusema mara moja kwa wiki, mara moja kwa mwezi,mara moja katika miezi kadha, mara moja, au hujawahi ?*

1=ONCE A WEEK
2=ONCE A MONTH
3=ONCE EVERY COUPLE OF MONTHS
4=ONCE
5=NEVER (**GO TO Q326)**

325. In the past 6 months did you use your savings to pay for any of the following? / *Je, katika miezi sita iliyopita, ulitumia akiba yako kulipia cho chote kati ya vifuatavyo?* **(READ ALL ALOUD. CIRCLE IF YES)**

1=FOOD FOR YOURSELF/ *chakula chako binafsi*
2=FOOD FOR YOUR FAMILY/ *chakula cha familia*
3=SCHOOL FEES FOR YOURSELF/ *karo yako ya shule*
4=SCHOOL SUPPLIES FOR YOURSELF/ *vitu vyako vya shule*
5=SCHOOL FEES FOR SOMEONE ELSE/ *karo ya shule ya mtu mwingine*
6=TRANSPORT/ *usafiri/nauli*
7=GIFTS/ *zawadi*
8=SANITARY TOWELS / *sodo/mlembe*
9=CLOTHES OR PERSONAL CARE (I.E. HAIR, LOTION, MAKEUP, ETC.) / *nguo au utunzi wa nafsi* (i.e. nywele, mafuta, mapambo, nk.)
10=MEDICAL EXPENSES FOR MYSELF / *matibabu yangu mimi mwenyewe*

11=MEDICAL EXPENSES FOR SOMEONE ELSE / *matibabu ya mtu mwingine*
96=OTHER (SPECIFY) / *mengine (taja)*

326. Who decides how you will use your savings – you alone, you together with someone else, or someone else? / *Je, ni nani anayeamua jinsi utakavyotumia akiba yako - wewe mwenyewe, wewe pamoja na mtu mwingie, ama mtu mwingine?*

1=YOU ALONE – *Wewe mwenyewe* **(GO TO Q328)**
2=YOU TOGETHER WITH SOMEONE ELSE – *Wewe pamoja na mtu mwingine*
3=SOMEONE ELSE – *Mtu mwingine*

327. Who is that other person? / *Je, ni nani huyo mtu mwingine?* **(CIRCLE ALL THAT APPLY. PROBE 'ANYONE ELSE?')**

1=MOTHER - *Mama*
2=FATHER - *Baba*
3=OTHER GUARDIAN – *Mlezi mwingine*
4=SISTER – *Dada yako*
5=BROTHER – *Ndugu yako*
6=OTHER NON-GUARDIAN RELATIVE – *Mtu wa jamii*
7=BOYFRIEND/HUSBAND – *Mpenzi au mume wako*
8=FRIEND - *Rafiki*
9=EMPLOYER - *Mwajiri*
96=OTHER (SPECIFY) ____________________________

328. Who do you think will control the money that you plan to save in the coming six months, you alone, you and someone else, or someone else? / *Je, unafikiri ni nani atakaye simamia akiba utakayoweka katika miezi sita ijayo, wewe mwenyewe, wewe pamoja na mtu mwingie ama mtu mwingine?*

1=YOU ALONE – *Wewe mwenyewe* **(GO TO Q401)**
2=YOU TOGETHER WITH SOMEONE ELSE – *Wewe pamoja na mtu mwingine*
3=SOMEONE ELSE – *Mtu mwingine*

329. Who is that other person? *Je, ni nani huyo mtu mwingine?* **(CIRCLE ALL THAT APPLY. PROBE 'ANYONE ELSE?')**

1=MOTHER - *Mama*
2=FATHER - *Baba*
3=OTHER GUARDIAN – *Mlezi mwingine*
4=SISTER – *Dada yako*
5=BROTHER – *Ndugu yako*
6=OTHER NON-GUARDIAN RELATIVE – *Mtu wa jamii*
7=BOYFRIEND/HUSBAND – *Mpenzi au mume wako*
8=FRIEND - *Rafiki*
9=EMPLOYER - *Mwajiri*
96=OTHER (SPECIFY) ____________________________

400. FINANCIAL LITERACY KNOWLEDGE AND ATTITUDES

Now I am going to ask you a few questions about money, how you use it or maybe plan for it. *Sasa nitakuuliza maswali kuhusu pesa; jinsi unavyoitumia au hata kuipangia.*

401. If I have one of each of the following coins in my pocket – 1/, 5/, 10/, 20/ - how much money do I have? / *Ikiwa nitakuwa na mojawapo ya shilingi zifuatazo – 1/, 5/, 10/, 20/ - Je, nitakuwa nina pesa ngapi mfukoni?*

(CORRECT RESPONSE IS KSH 36/)

1= CORRECT
2= INCORRECT
88=DON'T KNOW

402. If I have one of each of the following notes in my pocket – 50 KSh, 100 KSh, 200 KSh, 500 KSh, and 1000 KSh – how much money do I have? *Ikiwa nitakuwa na noti zifuatazo mfukoni mwangu – 50Ksh, 100Ksh, 200Ksh, 500Ksh, na 1000Kshs – Je, nitakuwa nina pesa ngapi?*

(CORRECT RESPONSE IS KSH 1850/)

1= CORRECT
2= INCORRECT
88=DON'T KNOW

403. Do you have a specific financial goal, for example saving to buy something that you would like, that you plan to achieve **one year from now**? / *Je, una lengo la pesa ulizo nazo, kwa mfano unaweka akiba ili ununue kitu fulani, ambacho ungependa kuwa nacho kwa* ***mwaka mmoja ujao?***

1=NO **(GO TO Q405)**
2=YES

404. Please describe what that goal is: / *Tafadhali eleza hilo lengo lako ni gani:*

____

____

____

405. Do you have a specific financial goal that you would like to achieve in the **next 3 months**? / *Je, una lengo la pesa ulizonazo ambalo ungependa kutimiza kwa* ***miezi mitatu ijiayo?***

1=NO **(GO TO Q407)**
2=YES

406. Please describe what that goal is: / *Tafadhali eleza hilo lengo lako ni gani:*

____

____

____

407. Do you have a plan for saving your money? / *Je, una mpango wa kuweka akiba ya pesa zako?*

1=NO **(GO TO Q410)**
2=YES

408. Is that plan written down? */ Je, umeandika chini mpango huo?*

1=NO
2=YES

409. What are two parts of that savings plan? / Taja sehemu mbili za hio mpango wa kuweka akiba*?*
(PROBE 'ANYTHING ELSE?' CIRCLE FIRST TWO RESPONSES.)

1= TOTAL SUM NEEDED
2= DATE LUMP SUM NEEDED BY
3= AMOUNT NEEDED TO SAVE PER WEEK/MONTH
4= NUMBER OF WEEKS/MONTHS NEEDED TO REACH TOTAL SUM
5= NONE OF THE ABOVE

410. Do you have a plan or budget for how to spend your money? / *Je, unampangilio/bajeti ya jinsi ya kutumia pesa zako?*

1=NO **(GO TO Q414)**
2=YES

411. Is that plan/budget written down? / *Je, umeandika chini mpango/bajeti huo?*

1=NO
2=YES

412. What are two sections of that plan/budget? / *Je, ni sehemu gani mbili za mpango/bajeti hio?*
(CIRCLE ALL THAT APPLY. PROBE 'ANYTHING ELSE?')
1= INCOME (MONEY EARNED)
2= EXPENSES (AMOUNT/THINGS SPENT MONEY ON)
3= NONE OF THE ABOVE

413. Would you say that you follow your spending plan all of the time, some of the time, rarely, or never? / *Je, unaweza kusema kwamba unafuata mpango wako wa matumizi ya pesa zako kila wakati, wakati mwingine, mara chache, sifuati kamwe?*

1=ALL OF THE TIME – *Kila wakati*
2=SOME OF THE TIME – *Wakati mwingine*
3=RARELY – *Mara chache*
4=NEVER – *Sifuati kamwe*

414. How important is it to you to save, would you say very important, somewhat important, or not important at all? / *Je, ni muhimu kwako kuweka akiba, unaweza kusema ni muhimu, muhimu kiasi, siyo muhimu?*

1=VERY IMPORTANT – *Ni muhimu*
2=SOMEWHAT IMPORTANT – *Muhimu kiasi*
3=NOT IMPORTANT AT ALL – *Si muhimu*

415. What are two reasons that saving money is important? / *Je, ni sababu gani mbili ambazo zinakufanya ufikirie ni muhimu kuweka akiba?* **(PROBE 'ANYTHING ELSE?' CIRCLE THE FIRST TWO RESPONSES)**

1= PERSONAL USE (clothes, entertainment, pads, etc)
2= EMERGENCIES
3= FUTURE OPPORTUNITIES (education/training, business, assets like a house, etc)
4= NONE OF THE ABOVE

416. What is one **informal** place to save money? / Taja *mahali pamoja pasio rasmi pa kuweka akiba?* **(CIRCLE THE FIRST OPTION MENTIONED)**

1= HOME BANK
2= HOLE IN THE GROUND
3= IN A BOX
4= MATTRESS
5= SUITCASE
6= WITH A FRIEND
7= WITH A PARENT OR RELATIVE
8= MERRY-GO-ROUND/CHAMA
9= NONE OF THE ABOVE

417. What is one **formal** way to save money? / *Taja njia moja rasmi ya kuweka akiba?* **(CIRCLE THE FIRST OPTION MENTIONED)**

1= BANK
2= SAVINGS ACCOUNT
3= MPESA
4= NONE OF THE ABOVE

418. What are two services or products you can get at a bank? / *Je, ni huduma gani mbili ambazo unaweza kupata kwenye benki?* **(PROBE 'ANYTHING ELSE?' CIRCLE THE FIRST TWO RESPONSES**)

1= LOAN
2= SAVINGS ACCOUNT
3= DEPOSIT
4= WITHDRAWALS
5= BANK CHEQUE
6= MONEY TRANSFERS
7= ATM SERVICES
8=FOREIGN EXCHANGE
9= NONE OF THE ABOVE

419. Have you ever been inside a bank? / *Je, umewahi kuingia ndani ya benki?*

1=NO
2=YES

420. Have you ever used a bank's services yourself? / *Je, umewahi kutumia huduma inayotolewa na benki?*

1=NO
2=YES

420b. Does anyone in the household you live in use a bank's services? / *Je, kunaye yeyote mnayeishi naye ambaye anatumia huduma za benki?*

1=NO
2=YES
88=DON'T KNOW

421. When someone takes a loan, what are two of her responsibilities? / *Je, ni majukumu gani mawili ambayo mtu huwa nayo wakati anapochukua mkopo kwa benki?* **(PROBE 'ANYTHING ELSE?' CIRCLE THE FIRST TWO RESPONSES**)

1= RETURN THE MONEY
2= PAY INTEREST ON THE LOAN
3= MAKE PAYMENTS ON TIME
4= MAKE LOAN REPAYMENTS
5= PAY BACK THE COST OF THE LOAN
6= NONE OF THE ABOVE

422-425. Now I'm going to ask you a few questions about your use of different financial services. Think about any financial services you may have used **before today**. If you are joining a savings group for the first time today, please do not count it. Have you ever used or participated in any of the following? / *Sasa nitakuuliza maswali machache kuhusu unavyotumia huduma tofauti za kifedha. Hebu fikiria huduma ye yote ambayo umewahi kutumia* ***kabla ya siku ya leo.*** *Ikiwa unajiunga kwa mara ya kwanza na kikundi cha kuweka akiba, tafadhali usihesabu kundi hilo.Je, umewahi kutumia au kushiriki katika mojawapo ya mambo yafuatayo?*

422. Group Savings Account with a Bank / *Akaunti ya pamoja ya benki*	1=NO 2=YES
423. Individual Savings Account with a Bank / *Akaunti ya kibinafsi ya benki*	1=NO 2=YES
424. Chama/Merry-Go-Round / *Chama*	1=NO 2=YES
425. Loan from a micro-finance organization or a bank / *Mkopo kutoka kwa Shirika la kutoa mikopo au benki*	1=NO 2=YES

426-433. Now I'm going to read you a story and then ask you a couple of questions about it.

Imani is 17 years old and lives with her mother and younger sister. Her older sister, Mary, is married and lives in another town, 3 hours away. Mary just had a baby boy, and Imani is eager to visit, to spend time with the baby, and help her sister.

Imani will need to save money for transport and a small gift for the baby. A cute little hat would be perfect! But she can't take money from her savings for this trip because she is saving that money to start her own business. Imani's dream is to start a small catering business. Hopefully, her neighbor will employ her to work extra days in her hotel so she can get the money she needs for her trip.

Sasa nitakusomea hadithi halafu baadaye nitakuuliza maswali kadhaa kuihusu.

Imani ana umri wa miaka 17 na anaishi na mama yake na dada yake mdogo. Dada yake mkubwa, Mary, ameolewa na anaishi katika jiji jingine, umbali wa masaa matatu. Mary amejifungua tu mtoto wa kiume, na Imani anatamani sana kumtembelea, ili achukue muda na mtoto huyo, na kumsaidia dada yake.

Imani atahitajika kuweka akiba ili apate nauli pamoja na pesa za kumnunulia mtoto zawadi. Kofia ndogo nzuri ni mwafaka! Lakini hawezi kuchukua pesa kutoka kwa hazina/akiba yake kwa sababu ya safari hii kwa sababu anaweka hazina ya kuanzisha biashara yake binafsi. Ndoto ya Imani ni kuanza biashara ndogo ya upishi. Ana matumaini kuwa , jirani yake ataweza kumuajiri kufanya kazi kwa siku kadhaa zaidi katika hoteli yake ili aweze kupata pesa anazozihitaji za usafiri wake.

426. What is one of Imani's short term financial goals: / *Mojawapo ya lengo la kifedha la Imani analohitajika kushughulilkia katika muda mfupi ni lipi:*

1=TRANSPORT TO VISIT HER SISTER
2=BUY A GIFT FOR HER NEPHEW
3=OPEN A CATERING BUSINESS
88=DON'T KNOW

427. What is Imani's long term financial goal: / *Lengo la Imani la kifedha ambalo anahitajika kushughulikia baadaye ni gani?*

1=TRANSPORT TO VISIT HER SISTER
2=BUY A GIFT FOR HER NEPHEW
3=OPEN A CATERING BUSINESS
88=DON'T KNOW

428. When someone opens a savings account at the bank, is that a formal or informal way to save? / *Mtu anapofungua akaunti ya akiba/hazina katika benki, je, hiyo ni njia rasmi au isiyo rasmi ya kuweka hazina/akiba?*

1=FORMAL
2=INFORMAL
88=DON'T KNOW

429. When someone keeps money in a tin in their house, is that a formal or informal way to save? / *Mtu anapoweka hazina katika mkebe ndani ya nyumba yao, je, hiyo ni njia rasmi au isiyo rasmi ya kuweka hazina/akiba?*

1=FORMAL
2=INFORMAL
88=DON'T KNOW

430. Grace would like to buy a new notebook for the next school term, which starts in eight weeks. If the notebook costs 500 Ksh and she can save 100 Ksh each week, will she reach her goal? */ Grace angependa kununua kijitabu kipya cha muhula ujao wa shule utakaoanza wiki nane zinazokuja. Iwapo kitabu kitagharimu shilingi 500 na anaweza kuhifadhi shilingi 100 kila wiki, je atahitimu/ atafikia lengo lake?*

1=NO
2=YES

431. In the situation I described in the previous question, if Grace figured out how much she needed to save each week in order to reach her goal. What would that be called: a budget, a savings plan, or a financial goal? / *Katika hali ambayo nimeeleza katika swali la awali, ikiwa Grace alitambua kiasi cha pesa ambacho atahitaji kuhifadhi katika kila wiki ili afikie lengo lake. Hiyo ingeweza kuitwaje: bajeti, mpango wa kuweka akiba, au lengo la pesa?*

1=BUDGET
2=SAVINGS PLAN
3=FINANCIAL GOAL
88=DON'T KNOW

432. Each week, Anna sits down and plans what she will earn and spend in the next week. She writes down all the places where she will get money and all the things she will spend it on. Then she is able to see if she has enough money for all of what she wants to buy. Anna has made a: budget, a savings plan, or a financial goal? *Kila wiki Anna hukaa chini na kupanga pesa ambazo atapata na atakazotumia katika wiki itakayofuata. Yeye huandika chini pahali pote ambapo atapata pesa, na kila kitu ambacho atatumia pesa hizo kwacho. Hii humwezesha kuona ikiwa ana pesa za kutosha kununulia vitu vyote ambavyo anataka kununua. Ann tayari ametengeneza: bajeti, mpango wa kuweka akiba, au lengo la pesa*

1=BUDGET
2=SAVINGS PLAN
3=FINANCIAL GOAL
88=DON'T KNOW

433. Do you agree or disagree –“Only people with a lot of money can save*”? / Je, unakubali au unakana – Ni watu walio na pesa nyingi pekee ndio wanaoweza kuweka akiba?*

1=AGREE
2=DISAGREE

500. SOCIAL NETWORKS/VULNERABILITY/GENDER

Now I’m going to ask you some questions about your friends. *Sasa nitakuuliza maswali kuhusu marafiki zako.*

501. How many friends do you have? /*Je, una marafiki wangapi?*

________________________ NUMBER OF FRIENDS (**IF NONE/ZERO GO TO Q508)**

502. How many close friends do you have, meaning people with whom you share your most personal secrets or concerns? /*Je, una marafiki wangapi wa karibu, yaani marafiki ambao unaweza kuwaeleza mambo yako ya siri ama yanayo kutia wasiwasi?*

__________________________ NUMBER OF CLOSE FRIENDS

503. How many of these friends could you count on if you needed to borrow money? / *Je, ni wangapi wa marafiki hawa ambao unaweza kutegema kama unahitaji kuomba msaada wa pesa?*

__________________________ NUMBER OF FRIENDS

504. How many of these friends could you count on if you had a problem or in case of an emergency? / Je, ni wangapi wa marafiki hawa ambao unaweza kutegemea ukiwa na shida ama jambo la dharura?

__________________________ NUMBER OF FRIENDS

505. Have you made any new friends in the past one year? / *Je, umepata marafiki wapya kwa mwaka moja uliopita?*

1=NO **(GO TO Q507)**
2=YES

506. How many friends did you make? / Je, *ulipata marafiki wangapi?*

__________________________ NUMBER OF FRIENDS

507. Other than your house, your friends’ house, or your school, where do you usually meet your girl friends? / Kando na nyumbani, nyumba la rafiki, au shule, *wewe hukutana wapi na marafiki zako wasichana*? **(PROBE ANYWHERE ELSE-CIRCLE ALL THAT APPLY)**

1= NEIGHBOURHOOD - *Mtaani*
2= HOTEL/RESTAURANT – *hoteli/mkahawa*
3= CHURCH/MOSQUE – *Kanisani / Msikitini*
4= YOUTH GROUP – *Kwenye makundi ya vijana*
5= WOMEN’S GROUP – *Kwenye makundi ya akina mama*
6= MARKET - *Sokoni*
7= RECREATIONAL CENTRE – *Kwenye sehemu za starehe*
8= WORKPLACE - *Kazini*
96=OTHER (SPECIFY) ____

Subject ID Number _____________

Now I want to ask you some questions about your surroundings and where you live. I will read a series of statements about friends and your neighborhood. For each of the statements please tell me if you agree or disagree. / *Sasa, nataka nikuulize maswali kuhusu mazingira yako na mahali unapoishi. Nitasoma sentenzi kuhusu marafiki na mtaa wako. Kwa kila mojawapo utasema ikiwa unakubaliana nayo ama unakataa.*

508. I have many friends in my neighborhood/community / *Nina marafiki wengi mtaani*	1=AGREE 2=DISAGREE
509. I feel safe walking around in my neighborhood/community during the day / *Huwa ninajihisi salama ninapotembea mtaani wakati wa mchana*	1=AGREE 2=DISAGREE
510. I feel safe walking around in my neighborhood/community after dark / *Huwa najihisi salama ninapotembea mtaani wakati wa usiku*	1=AGREE 2=DISAGREE
511. There is a lot of crime in my neighborhood/community / *Kuna uhalifu mwingi mtaani*	1=AGREE 2=DISAGREE
512. I would be much happier if I lived in another community / *Ningefurahi kama ningeishi katika mtaa mwingine*	1=AGREE 2=DISAGREE
513. People in my neighborhood trust one another / *Watu wanaoishi katika mtaa wangu wanaaminiana*	1=AGREE 2=DISAGREE
514. I know girls in my neighborhood that have been raped / *Ninajua wasichana wa mtaa wangu ambao wamewahi kunajisiwa*	1=AGREE 2=DISAGREE
515. At times, I feel scared that I will be raped / *Wakatimwingine huwa ninahofu kwamba huenda nikanajisiwa*	1=AGREE 2=DISAGREE
516. In the past six months, I have been touched indecently by someone of the opposite sex in my neighborhood / *Katika miezi sita iliyopita nimewahi kushikwa shikwa vibaya na mtu tunaoishi nao mtaani wa jinsia tofauti*	1=AGREE 2=DISAGREE
517. I have been robbed in the past six months / *Nimewahi kuibiwa kwa kipindi cha miezi sita iliyopita*	1=AGREE 2=DISAGREE
518. In my neighborhood, people of the opposite sex tease me as I go about my day / *Kuna watu wa jinsia tofauti mtaani ambao hunichokoza ninapoendelea na shughuli zangu*	1=AGREE 2=DISAGREE
519. There is a female adult in my community, other than my parents or teacher, who I can turn to if I had a serious problem / *Kuna mwanamke katika mtaa wangu, kando na wazazi wangu ama mwalimu ambaye ninaweza kumwendea wakati wa shida*	1=AGREE 2=DISAGREE
520. There is a female adult, that is not my mother or teacher, who I meet regularly in my life that I can discuss my problems, joys, and ask questions of / *Kuna mwanamke, kando na mama yangu ama mwalimu, ambaye ninakutana naye kila mara kujadiliana naye juu ya shida zangu, furaha na kumwuliza maswali*	1=AGREE 2=DISAGREE

521. Can you count on the following people if you needed money urgently? / *Ungeweza kutegemea watu wafuatao ukihitaji pesa kwa haraka?* **(READ THE LIST. IF NO ON Q521A GO TO THE NEXT ITEM, SKIP Q521B, Q521C)**

	521A.	521B. SEX Male/Female	521C. Residence Location
A. PARENT/GUARDIAN / *mzazi/mlezi*	1= NO 2= YES	1=Male 2=Female	
B. SIBLING/*ndugu/dada*	1= NO 2= YES	1=Male 2=Female	
C. RELATIVE/*mtu wa jamii*	1= NO 2= YES	1=Male 2=Female	
D. A CLOSE FRIEND/ *rafiki wa karibu*	1= NO 2= YES	1=Male 2=Female	
E. NEIGHBOR/*jirani*	1= NO 2= YES	1=Male 2=Female	
F. BOYFRIEND/HUSBAND/*mpenzi wa kiume/mume*	1= NO 2= YES	1=Male 2=Female	
G. MENTOR/ *Mshauri au Mwongozi*	1= NO 2= YES	1=Male 2=Female	
H. TEACHER/ *mwalimu*	1= NO 2= YES	1=Male 2=Female	

Residence Location Codes:

1=Resides in my household
2=Resides in this community
3=Resides in neighboring community
4=Resides upcountry (rural area)

522. Other than your parent or guardian, could you count on the following people to take you in for the night in case of an emergency? / *Bali na wazazi/mlezi, ungeweza kutegemea watu wafuatao kukupatia malazi ukiwa na jambo la dharura?* **(READ THE LIST. IF NO ON Q522A GO TO THE NEXT ITEM, SKIP Q522B, Q522C)**

	522A.	522B. SEX Male/Female	522C. Residence Location
A. SIBLING/*ndugu/dada*	1= NO 2= YES	1=Male 2=Female	
B. RELATIVE/*mtu wa jamii*	1= NO 2= YES	1=Male 2=Female	
C. A CLOSE FRIEND/ *rafiki wa karibu*	1= NO 2= YES	1=Male 2=Female	
D. NEIGHBOR/*jirani*	1= NO 2= YES	1=Male 2=Female	
E. BOYFRIEND/HUSBAND/*mpenzi wa kiume/mume*	1= NO 2= YES	1=Male 2=Female	
F. MENTOR/ *Mshauri au Mwongozi*	1= NO 2= YES	1=Male 2=Female	
G. TEACHER/ *mwalimu*			

Residence Location Codes:

1=Resides in my household
2=Resides in this community
3=Resides in neighboring community
4=Resides upcountry (rural area)

For each of the following places, please tell me whether you are usually permitted to go to them on your own, only if someone accompanies you, or not at all: / *Tafadhali niambie kama unaruhusiwa kwenda sehemu zifuatazo ukiwa peke yako, pamoja na mtu mwingine, ama hauruhusiwi kamwe:*

523. Local Market / *Sokoni*	1=ON MY OWN<br>2=IF SOMEONE ACCOMPANIES ME<br>3=NOT AT ALL<br>9=NEVER GO THERE
524. Local Health Clinic / *Hospitalini*	1=ON MY OWN<br>2=IF SOMEONE ACCOMPANIES ME<br>3=NOT AT ALL<br>9=NEVER GO THERE
525. Homes of friends in my neighborhood / *Nyumbani mwa marafiki mtaani*	1=ON MY OWN<br>2=IF SOMEONE ACCOMPANIES ME<br>3=NOT AT ALL<br>9=NEVER GO THERE
526. School / *Shuleni*	1=ON MY OWN<br>2=IF SOMEONE ACCOMPANIES ME<br>3=NOT AT ALL<br>9=NEVER GO THERE
527. Work/Business / *Kazini*	1=ON MY OWN<br>2=IF SOMEONE ACCOMPANIES ME<br>3=NOT AT ALL<br>9=NEVER GO THERE
528. Youth Group/Girls Group / *Kwenye makundi ya vijana/wasichana*	1=ON MY OWN<br>2=IF SOMEONE ACCOMPANIES ME<br>3=NOT AT ALL<br>9=NEVER GO THERE

Now let's talk about your situation in your household and how you feel about yourself. I will read a series of statements, please tell me if it applies to you – in other words, if you agree or disagree. / Hebu sasa tuzungumzie hali ya nyumbani kwenu na jinsi unavyo ichukulia. *Nitasoma sentensi kadhaa, tafadhali niambie ikiwa zinakuzungumzia, yaani kama unakubaliana nazo ama unakataa.*

529. You need someone's permission before you leave the house / *Unahitaji ruhusa kabla hujatoka nyumbani*	1=AGREE 2=DISAGREE
530. You need someone's permission before you visit a friend / *Unahitaji ruhusa ya kutoka kabla hujatembelea rafiki*	1=AGREE 2=DISAGREE
531. You need someone's permission before you spend your money / *Unahitaji ruhusa kabla ya kutumia pesa zako.*	1=AGREE 2=DISAGREE
532. You need someone's permission before you look for a job */ Unahitaji ruhusa kabla ya kutafuta kazi*	1=AGREE 2=DISAGREE

533. You feel as intelligent as most other people your age / *Unajihisi kuwa mwerevu kama vijana wengine wa umri wako*	1=AGREE 2=DISAGREE
534. You sometimes feel worthless / *Kuna wakati wewe hujihisi kuwa hustahili*	1=AGREE 2=DISAGREE
535. You don't have hope for your future / *Wewe huna tumaini la siku zako za usoni*	1=AGREE 2=DISAGREE
536. You think you have/had good parents/guardians / *Unadhani kuwa wazazi/walezi wazuri*	1=AGREE 2=DISAGREE
537. You are optimistic that you will have a better life than your parents/guardians / *Una matumaini kuwa utakuwa na maisha bora kuliko ya wazazi/walezi wako*	1=AGREE 2=DISAGREE
538. You wish your parents/guardians placed more value on your education /	1=AGREE 2=DISAGREE

Unatamani wazazi/walezi wako wangedhamini zaidi elimu yako	
539. You feel you make good decisions concerning how to manage your money / *Unadhani kwamba huwa unafanya uamuzi mzuri kuhusu jinsi ya kutumia pesa zako.*	1=AGREE 2=DISAGREE
540. You always manage to solve difficult problems if you try hard enough./ *Kila mara unaweza kutatua shida/matatizo magumu ukitia mkazo zaidi.*	1=AGREE 2=DISAGREE
541. If someone is against you, you can still find ways to get what you want./ *Kama mtu anakudhulumu, bado unaweza kupata njia za kupata kileunachohitaji*	1=AGREE 2=DISAGREE
542. It is easy for you to focus on your aims and accomplish your goals. / *Ni rahisi kwako kufuata nia zako na kukamilisha mipango zako*	1=AGREE 2=DISAGREE
543. You are confident that you could handle unexpected events very well./ *Unaamini ya kwamba unaweza tatua maswala yasiyotarajiwa vizuri sana*	1=AGREE 2=DISAGREE
544. Because of the help you can get, you know how to manage unexpected situations./ *Kwa sababu ya usaidizi unaweza pata, unajua kutatua maswala yasiyotarajiwa*	1=AGREE 2=DISAGREE
545. You can solve most problems if you make the necessary effort./ *Unaweza sahihisha matatizo mengi ukitia bidii vilivyo*	1=AGREE 2=DISAGREE
546. You can remain calm when facing difficulties because you can rely on your own abilities./ *Unaweza kutulia ukikumbwa na matatizo kwa sababu unaweza tegemea uwezo wako binafsi*	1=AGREE 2=DISAGREE
547. When you face a problem, you can usually find more than one solution./ *Ukiwa na tatizo unaweza pata suluhisho zaidi ya moja*	1=AGREE 2=DISAGREE
548. If you are in trouble, you can usually think of a solution./*Ukiwa na shida huwa unaweza fikiria suluhisho*	1=AGREE 2=DISAGREE
549. You can usually handle any situation that comes your way./ *Huwa unaweza kushugulikia hali yeyote inayojitokeza*	1=AGREE 2=DISAGREE

Now let's talk about the roles of women and men. I will read a series of statements. Please tell me if you agree or disagree. / *Sasa, nataka tuzungumze kuhusu majukumu za wanawake na wanaume. Nitasoma sentensi kadhaa. Tafadhali niambie kama unakubaliana nazo ama unakataa*

550. Girls are not as good as boys in school / *Wasichana hawafanyi vizuri shuleni kama wavulana.*	1=AGREE 2=DISAGREE
551. When money is scarce and parents cannot send all children to school, boys should be sent before girls / *Wakati kuna upungufu wa pesa na wazazi hawana namna ya kusomesha watoto wote, ni vyema wavulana wapelekwe shuleni kabla ya wasichana.*	1=AGREE 2=DISAGREE
552. Some girls who are raped deserve it because of the way they dress or talk to boys / *Baadhi ya wasichana wanaonajisiwa wanastahili kwa sababu ya mavazi yao ama jinsi wanavyo zungumza na wavulana.*	1=AGREE 2=DISAGREE
553. Boys should do as much domestic work as girls / *Wavulana wanastahili kufanya kazi za nyumbani sawasawa na wasichana.*	1=AGREE 2=DISAGREE
554. Girls can make as good leaders as boys / *Wasichana wanaweza kuwa viongozi wema kama wavulana*	1=AGREE 2=DISAGREE

555. Men who force girls to have sex should be sent to jail / *Wanaume ambao hulazimisha wasichana kufanya mapenzi wanapaswa kufungwa jela*	1=AGREE 2=DISAGREE
556. Men rape girls because they can't control themselves / Wanaume *wananajisi wasichana kwa sababu hawawezi kujizuia.*	1=AGREE 2=DISAGREE
557. Boys should not be asked to help their mothers prepare food / *Wavulana hawastahili kuulizwa wasaidie mama zao kupika chakula.*	1=AGREE 2=DISAGREE

Now let's talk about **work and other money issues**. I will read a series of statements. Please tell me if you agree or disagree. / *Sasa, tuzungumze kuhusu kazi na pesa. Nitasoma sentensi kadhaa. Tafadhali niambie kama unakubaliana nazo ama unakataa.*

558. If you really needed the money, you would stay at a job where the boss abuses you. / *Ikiwa unahitaji pesa sana, utafanya kazi hiyo hata kama mkubwa wako anakudhulumu*	1=AGREE 2=DISAGREE
559. Men are better at managing money than women. / *Wanaume wanajua kutunza pesa vizuri kuliko wanawake.*	1=AGREE 2=DISAGREE
560. Fathers in the family should decide on how family money is spent. / *Akina baba ndio wanastahili kuamua jinsi ya kutumia pesa nyumbani.*	1=AGREE 2=DISAGREE
561. Husbands should be allowed to stop their wives from working. / *Wanaume wanastahili kukubaliwa kuwakataza wake zao kufanya kazi.*	1=AGREE 2=DISAGREE
562. Bank accounts are for rich people. / *Akaunti za benki ni za matajiri*	1=AGREE 2=DISAGREE

600. HIV/AIDS

Now I'm going to ask you some questions about what you know about HIV/AIDS and other reproductive health issues. Some girls know a lot about these issues, others know very little. Just answer as best as you can. *Sasa nitakuuliza maswali kuhusu unavyoelewa virusi vya ukimwi na mambo mengine yanayohusiana na afya ya uzazi. Wasichana wengine wanaelewa sana kuhusu mambo haya ilihali wengine hawaelewi sana. Jibu tu kwa kadri ya uwezo wako*.

601. I would like to ask you some questions about HIV. I will read a series of statements about HIV/AIDS and I want you to tell me if you agree or disagree with the statement. *Nataka nikuulize maswali kuhusu virusi vya UKIMWI . Nitasoma sentensi kadhaa kuhusu UKIMWI na ningetaka uniambie ikiwa unakubaliana au hukubaliani na semi hizo.*

	**1= AGREE**	**2 = DISAGREE**	**88 = DK**
A. One can get HIV from mosquito bites / *Mtu anaweza kupata virusi vya UKIMWI kwa kuumwa na mbu*	1	2	88
B. Women can give HIV to their children through their breast milk / *Wanawake wanaweza kuambukiza watoto wao virusi vya UKIMWI kwa kuwanyonyesha*	1	2	88
C. There is a way that mothers with HIV can protect their unborn children from HIV / *Kuna njia ambayo wamama walio na UKIMWI*	1	2	88

wanaweza kuwalinda watoto kutopata virusi vya UKIMWI			
D. A healthy looking person can be infected with HIV / *Mtu mwenye afya nzuri anaweza Kuwa na virusi vya UKIMWI*	1	2	88
E. People only contract HIV/AIDS in large towns and cities / *Watu wanaoambukizwa virusi vya UKIMWI wako katika miji mikubwa pekee*	1	2	88
F. Most people get AIDS from accidents with sharp instruments / *Watu wengi hupata ugonjwa wa UKIMWI kutokana na ajali ya kutumia vyombo vikali (sindano na wembe)*	1	2	88
G. There is now medical treatment for people living with HIV, to improve their quality of life / *Kwa sasa kuna matibabu kwa watu wanaoishi na virusi ili kuimarisha afya yao*	1	2	88
H. One can get AIDS from sharing eating utensils such as knives and forks / *Mtu anaweza kuambukizwa UKIMWI kwa kula pamoja kwa kutumia visu na vijiko/uma*	1	2	88
I. There is now a cure for AIDS / *Kuna tiba ya UKIMWI sasa*	1	2	88
J. If one has other sexually transmitted infections, it increases one's chance of getting HIV / *Mtu mwenye magonjwa ya zinaa anaweza kuambukizwa kwa urahisi na UKIMWI*	1	2	88

602. Please mention all the ways in which a person can be infected with HIV? / *Tafadhali taja njia zote ambazo mtu anaweza kuambukizwa Virusi vya UKIMWI?* **(PROBE 'ANYTHING ELSE?' CIRCLE ALL THAT APPLY)**

1=SEXUAL INTERCOURSE - *Ngono*
2=INJECTIONS WITH UNSTERILIZED NEEDLES – *Kudungwa na sindano*
3=CIRCUMCISION WITH UNSTERILIZED TOOLS – *Kutahiriwa na vifaa visivyo safi*
4=DURING PREGNANCY/CHILDBIRTH - *Wakati wa mimba/Kuzaa*
5=THROUGH BREAST MILK – *Kwa kunyonyesha*
6=TRANSFUSION OF INFECTED BLOOD – *Kupewa damu yenye virusi*
7=SHARING FOOD UTENSILS – *Kutumia chombo kimoja (sahani, kikombe)*
8=THROUGH SEX WITH COMMERCIAL SEX WORKERS – *Kufanya mapenzi na kahaba*
9=HUGGING OR KISSING – *Kukumbatiana au Kubusiana*
10=RAPE – Ubakaji
11=SHARING SHARP OBJECTS – Kushirikiana vitu vyenye ncha kali
96=OTHER (SPECIFY) ____
88=DON'T KNOW - *Sijui*

603. Can a person do anything to protect him/herself from getting HIV? / *Je, mtu anaweza kujizuia asiweze kuambukizwa na virusi vya HIV?*

1=NO **(GO TO Q605)**
2=YES

604. How can people protect themselves from getting infected with HIV? / *Je, watu wanaweza kujizuia kwa njia gani wasiambukizwe virusi vya HIV?* **(PROBE 'ANYTHING ELSE?' CIRCLE ALL THAT APPLY)**

1=ABSTAIN FROM SEX – *Kujizuia kufanya ngono*
2=NON-PENETRATIVE SEX/THIGH SEX/DRY SEX – *Ngono ya kutoingiza ndani/ya Mpaja/Kutomwaga*

3=ALWAYS USE CONDOMS – *Kutumia kinga (kondomu) kila wakati*
4=LIMITED NUMBER OF SEX PARTNERS – *Kuwa na wapenzi wachache wa kufanya ngono*
5=HAVE ONLY ONE SEX PARTNER – *Kuwa na mpenzi mmoja*
6=AVOID SEX WITH COMMERCIAL SEX WORKERS – *Kuepuka ngono na makahaba*
7=HAVE SEX WITH A VIRGIN – *Kufanya ngono na bikira*
8=USE STERILIZED NEEDLES – *Kutumia sindano safi*
9=REQUIRE PARTNERS TO TAKE BLOOD TEST – *Kuwahimiza marafiki kuchunguzwa damu*
96=OTHER (SPECIFY) ____
88=DON'T KNOW – *Sijui*

605. Do you ever worry that you might be HIV positive? /*Je, huwa unashuku kwamba una virusi vya UKIMWI?*

1=NO
2=YES

606. Do you know where to get a test for HIV? / *Je, unajua mahali pa kupimia hali yako ya virusi ya HIV?*

1=NO
2=YES

607. Have you had an HIV test? / *Je, umewahi kupimwa hali ya virusi vya ukimwi?*

1=NO **(GO TO Q610)**
2=YES

608. What was the reason you were tested? *Je, kwa nini ulipimwa UKIMWI? (* ***ASK TWICE*** **'ANYTHING ELSE?' MORE THAN ONE RESPONSE POSSIBLE. CIRCLE ALL THAT APPLY)**

1 = WANTED TO KNOW STATUS/CURIOSITY
2 = BEFORE MARRIAGE
3 = BEFORE PREGNANCY/CHILDBIRTH
4 = WAS SICK
5 = WAS ADVISED BY FRIEND/RELATIVE
6 = WAS ADVISED BY HEALTHCARE PROVIDER
7 = PART OF ROUTINE PRENATAL CARE
96 = OTHER (SPECIFY) ……………………………………………

609. When was your most recent HIV test? *Je, ulipimwa UKIMWI mara ya mwisho lini?* (**IF LESS THAN ONE MONTH, WRITE '00'. 8888=DON'T KNOW)**

NUMBER OF MONTHS ____________________________ (**GO TO Q700)**

610. What is the main reason you have not been tested? *Je, sababu ipi kuu haujawahi kupimwa UKIMWI?*

1 = NOT AT RISK
2 = USE CONDOMS CONSISTENTLY
3 = DO NOT HAVE SEX
4 = HAVE ONE FAITHFUL PARTNER
5 = TRUST PARTNER
6 = DO NOT KNOW WHERE TO GET TESTED
7 = CANNOT AFFORD TEST/TRANSPORT TO TEST
8 = DON'T FEEL SICK
9 = AFRAID TO BE TESTED
96 = OTHER (SPECIFY*)* ………………………………………………

700. **FAMILY PLANNING AND PARENTAL COMMUNICATION**

701. Now I would like to talk to you about family planning – the various methods a couple can use to delay or avoid a pregnancy. Which ways or methods have you heard about?
Sasa ningependa kukuzungumzia kuhusu mpango wa uzazi – njia tofauti watu wawili wanaweza kutumia kuchelewesha au kuepuka mimba. Ni njia gani umewahi kuzisikia?

CIRCLE ALL METHODS SPONTANEOUSLY MENTIONED (CODE 2). READ THE LIST. IF RESPONDENT RECOGNIZES THE METHOD, CIRCLE CODE 3 (PROBED). IF NOT RECOGNIZED, CIRLCE CODE '1.'

LIST OF METHODS	1=NO	2 =YES, SPONT.	3= YES, PROBED
A. PILL – Women can take a pill every day./ *TEMBE– wanawake wanaweza kumeza tembe kila siku.*	1	2	3
B. IUCD – Women can have a loop or coil placed inside them by a doctor or nurse / *KOILI– wanawake wanaweza kuwekewa koili ndani yao na daktari au muuguzi*.	1	2	3
C. INJECTABLE/DEPO – Women can have an injection which stops them from becoming pregnant for several months / *SINDANO/DEPO – wanawake wanaweza kudungwa sindano ambayo huzuia kupata mimba kwa miezi kadhaa.*	1	2	3
D. VASECTOMY/TUBAL LIGATION – A man or a woman can have an operation to avoid having any more babies/ *KUKATWA MSHIPA WA KIUME/KIKE – mwanaume au mwanamke anaweza fanya operesheni kuzuia kupata watoto tena*	1	2	3
E. MALE CONDOM – Men can use a rubber sheath while playing sex / *KONDOMU YA KIUME – wanaume wanaweza kuvaa mpira wakifanya ngono*.	1	2	3
F. FEMALE CONDOM – A woman can place a rubber sheath in her vagina before intercourse/ *KONDOMU YA KIKE – mwanamke anaweza kuweka mpira ndani ya uke wake kabla ya ngono*	1	2	3
G. JADELLE/NORPLANT/IMPLANON® - Women can have small rods put under the skin in their arm. / *Wanawake wanaweza weka vijiti vidogo chini ya ngozi ya upande wa juu ya mkono.*	1	2	3
H. EMERGENCY CONTRACEPTION/ MORNING AFTER PILL. A lady takes pills or has an IUCD put in within 72 hours of unprotected sex. / TEMBE YA DHARURA YA KUPANGA UZAZI/TEMBE YA ASUBUHI BAADA YA KUFANYA NGONO BILA KINGA *mwanamke anaweza kunywa tembe ama kuwekwa koili kati ya masaa sabini na mbili baada ya kufanya ngono bila kinga.*	1	2	3
I. NATURAL F.P. A lady takes her temperature everyday or checks mucus to tell the days she is likely to get pregnant./ NJIA YA KAWAIDA YA KUPANGA UZAZI - *mwanamke hupima joto lake kila siku au kuangalia ute kujua zile siku angeweza kupata mimba.*	1	2	3
J. SAFE DAYS – A lady can count the number of days since her last period to tell when she is most likely to get pregnant./ SIKU ZA KINGA – *mke anaweza hesabu siku zake tangu apate hedhi yake ya mwisho ili kujua ni lini anaweza pata mimba.*	1	2	3
K. WITHDRAWAL – Men can be careful and pull out before climax./ *KUMWAGA NJE – wanaume wanaweza kuwa waangalifu nakutoka kabla ya kushusha.*	1	2	3
L. FOAMING TABLET, CREAM, JELLY – A woman can place a tablet, cream or jelly in her vagina before intercourse/ *TEMBE YA POVU, KRIMU, MAFUTA – Mwanamke anaweza weka tembe, krimu au mafuta ndani ya uke wake kabla ya ngono.*	1	2	3

702. I will read some statements about condoms and I would like you to tell me if you agree or disagree with the statement. / *Nitasoma baadhi ya kauli kuhusu kondomu na ningependa uniambie ikiwa unakubaliana au hukubaliani na semi hizo.* (**READ EACH STATEMENT AND ASK IF THE RESPONDENT AGREES OR DISAGREES)**

1= AGREE	2= DISAGREE	88= DK

A. A condom prevents pregnancy but not HIV / *Kondomu huzuia kupata mimba bali sio virusi vya UKIMWI.*	1	2	88
B. Using two condoms at the same time is better than using just one / *Kutumia kondomu mbili kwa wakati mmoja ni bora kuliko kutumia moja pekee.*	1	2	88
C. Most people who use condoms are promiscuous / *Watu wengi ambao hutumia kondomu si waaminifu.*	1	2	88
D. Condoms decrease intimacy or emotional closeness / *Kondomu hupunguza mapenzi au hisia za karibu.*	1	2	88
E. Condoms are effective in preventing HIV transmission / *Kondomu ni kinga ya kuzuia usambazaji wa virusi vya UKIMWI.*	1	2	88
F. I would refuse to have sex with someone who is not prepared to use a condom / *Nitakataa kufanya ngono na mtu ambaye hayuko tayari kutumia kondomu.*	1	2	88
G. Condoms should not be reused / *Kondomu hazistahili kutumiwa tena baada* **ya** *kutumika.*	1	2	88
H. A husband should not use condoms with his wife / *Mume hapaswi kutumia kondomu na mkewe.*	1	2	88
I. Moral people can use condoms / *Watu waadilifu/tabia nzuri wanaweza kutumia kondomu.*	1	2	88
J. Your religion forbids use of condoms / *Dini yako inakataza kutumia kondomu.*	1	2	88
K. It is a man's responsibility to provide the condom / *Ni jukumu la mwanaume kuwa/kupeana kondomu.*	1	2	88
L. I feel confident that I can insist on condom use every time I have sex / *Najiamini kuwa naweza kusisitiza kutimia kondomu kila wakati nikifanya ngono.*	1	2	88

703. Have you talked about reproductive/sexual health issues with your **mother/female guardian** in the last 6 months? / *Je, umewahi kuzungumzia na* ***mama yako/mama mlezi*** *kuhusu hali ya uzazi ama kufanya ngono au mapenzi miezi sita iliyopita?*

1=NO **(GO TO Q712)**
2=YES

704-711. Which of the following reproductive/sexual health issues have you discussed? / *Je, ni yapi kati ya mambo ya hali ya uzazi ama kufanya ngono au mapenzi yafuatayo ambayo umewahi kuzungumzia?*

704. Menstruation / *Kuona hedhi au damu ya mwezi*	1=NO 2=YES
. HIV/AIDS / *UKIMWI*	1=NO 2=YES
. STI's / *Magonjwa ya zinaa*	1=NO 2=YES
. Pregnancy / *Uja uzito au Kuwa na mimba*	1=NO 2=YES
. Family Planning/Contraception / *Kupanga uzazi*	1=NO 2=YES
. Marriage / *Ndoa*	1=NO 2=YES
. Sexual relationships / *Uhusiano wa kimapenzi*	1=NO 2=YES

705. Which other reproductive/sexual issues did you discuss? / *Je, ni nini ingine ulizungumzia kuhusu hali ya uzazi ama kufanya ngono au mapenzi? (TAJA)*

(SPECIFY) ____

9=NONE

712. Have you talked about reproductive/sexual issues with your **father/male guardian** in the last 6 months? / *Je, umewahi kuzungumzia na **baba yako/baba mlezi** kuhusu hali ya uzazi ama kufanya ngono au mapenzi miezi sita iliyopita?*

1=NO **(GO TO FILTER)**
2=YES

713-720. Which of the following reproductive/sexual health issues have you discussed? / *Je, ni yapi kati ya mambo ya hali ya uzazi ama kufanya ngono au mapenzi yafuatayo ambayo umewahi kuzungumzia?*

713. Menstruation / *Kuona hedhi au damu ya mwezi*	1=NO 2=YES
. HIV/AIDS / *UKIMWI*	1=NO 2=YES
. STI's / *Magonjwa ya zinaa*	1=NO 2=YES
. Pregnancy / *Uja uzito au kuwa na mamba*	1=NO 2=YES
. Family Planning/Contraception / *Kupanga uzazi*	1=NO 2=YES
. Marriage / *Ndoa*	1=NO 2=YES
. Sexual relationships / *Uhusiano wa kimapenzi*	1=NO 2=YES

714. Which other reproductive/sexual issues did you discuss? / *Je, ni nini ingine ulizungumzia kuhusu hali ya uzazi ama kufanya ngano au mapenzi? (TAJA)*

(SPECIFY) ____

9=NONE

FILTER: INTERVIEWER – CHECK Q122. DOES R LIVES WITH HUSBAND OR BOYFRIEND?
1=NO (GO TO Q730)
2=YES (ASK Q721-729)

721. Have you talked about reproductive/sexual issues with your **husband/boyfriend** in the last 6 months? / *Je, umewahi kuzungumzia na **mume/mpenzi wako** kuhusu hali ya uzazi ama kufanya ngono au mapenzi miezi sita iliyopita?*

1=NO **(GO TO Q730)**
2=YES

722-729. Which of the following reproductive/sexual health issues have you discussed? / *Je, ni yapi kati ya mambo ya hali ya uzazi ama kufanya ngono au mapenzi yafuatayo ambayo umewahi kuzungumzia?*

722. Menstruation / *Kuona hedhi au damu ya mwezi*	1=NO 2=YES

. HIV/AIDS / *UKIMWI*	1=NO 2=YES
. STI's / *Magonjwa ya zinaa*	1=NO 2=YES
. Pregnancy / *uja uzito au kuwa na mamba*	1=NO 2=YES
. Family Planning/Contraception / *Kupanga uzazi*	1=NO 2=YES
. Marriage / *Ndoa*	1=NO 2=YES
. Sexual relationships / *Uhusiano wa kimapenzi*	1=NO 2=YES

723. Which other reproductive/sexual issues did you discuss? / *Je, ni nini ingine ulizungumzia kuhusu hali ya uzazi ama kufanya ngano au mapenzi? (TAJA)*

(SPECIFY) ____

9=NONE

730. Do you discuss reproductive/sexual health issues with anyone else besides your mother/female guardian, father/male guardian, or husband/boyfriend? / *Je, huwa unazungumzia kuhusu mambo ya hali ya uzazi ama kufanya ngono au mapenzi na mtu mwingine bali na mama yako/mama mlezi, baba yako/baba mlezi, au mume/mpenzi wako?*

1=NO **(GO TO SECTION 800)**
2=YES

731. With who else do you discuss reproductive/sexual health issues? / *Je, ni nani mwingine ambaye huwa unazungumza naye kuhusu hali ya uzazi ama kufanya ngono au mapenzi?* **(READ EACH ITEM ALOUD. ASK YES OR NO)**

A. SISTER / *Dada yako*	1=NO 2=YES
B. BROTHER / *Ndugu yako*	1=NO 2=YES
C. AUNT / *Shangazi*	1=NO 2=YES
D. UNCLE / *Mjomba*	1=NO 2=YES
E. OTHER RELATIVE / Watu unaohusiana nao	1=NO 2=YES
F. FRIEND / *Rafiki*	1=NO 2=YES
G. MENTOR / *Mshauri au Mwongozi*	1=NO 2=YES
H. NON-LIVE IN BOYFRIEND / *Mpenzi wa kiume msiyeishi naye*	1=NO 2=YES
I. TEACHER / *mwalimu*	
J. OTHER / *nyingine*	1=NO 2=YES

732. Which other reproductive/sexual issues did you discuss? / *Je, ni nini ingine ulizungumzia kuhusu hali ya uzazi ama kufanya ngano au mapenzi? (TAJA)*

(SPECIFY) ____

9=NONE

800. SEXUAL BEHAVIOR AND REPRODUCTIVE HEALTH

Now I am going to ask you some questions about having sex. We are asking these questions to learn more about the experiences of girls like yourself and how you feel in order to make the lives of young people safer. We know that some girls have sex and some have sex with more than one person. Please try to answer the following questions honestly. Remember, your answers are strictly confidential. / *Sasa nitakuuliza maswali juu ya kufanya ngono. Tunauliza maswali haya ili tujue yale wasichana kama wewe mnafahamu na jinsi mnavyohisi ili tufanye maisha ya wasichana kuwa salama. Tunafahamu kwamba baadhi ya wasichana wanafanya ngono na wengine hufanya ngono na zaidi ya mpenzi mmoja. Tafadhali jibu maswali yafuatayo kwa uaminifu. Kumbuka kwamba majibu yako yatabakia kuwa siri..*

801. At what age did you first have sexual intercourse? / *Ni umri gani ulifanya ngono kwa mara ya kwanza?*

__________________________ AGE IN YEARS
97=NEVER HAD SEXUAL INTERCOURSE (**GO TO SECTION 900)**

802. How old was the person with whom you first had sex? / *Mtu mliyefanya naye ngono mara ya kwanza alikuwa na umri gani?* **(IF UNSURE, ASK RESPONDENT TO ESTIMATE)**

__________________________ AGE IN YEARS

803. On that first time did you or your partner do anything to avoid a pregnancy? *Je, wewe au mpenzi wako mlifanya kitu kuzuia mimba mara ya kwanza?*

1=NO (**GO TO Q805)**
2=YES

804. Which method did you use? / *Mlitumia njia gani?*

1=PILL / *Tembe au vidonge vya kupanga uzazi*
2=CONDOM – *Kondomu*
3=INJECTION – *Kudungwa sindano*
4=WITHDRAWAL – *Kutoa nje kabla ya kumwaga*
5=SAFEDAYS – *Kuhesabu siku baada ya kuwa na hedhi au kuona mwezi*
6=OTHER FORM OF CONTRACEPTION – *Aina nyingine ya kupanga uzazi*
7=NOTHING – *Sikufanya lo lote*
96=OTHER (SPECIFY) ________________________________

805. Did your partner give you any money or gifts or pay for something for you in the first month of your relationship? / *Je, mpenzi wako alikupatia pesa au zawadi au akakulipia kitu katika mwezi wa kwanza wa uhusiano wenu?*

1=NO (**GO TO Q807)**
2=YES

806. About how much did your partner spend in total on these in the first month of your relationship? / *Kwa jumla, mpenzi wako alitumia pesa ngapi katika mwezi wa kwanza wa uhusiano wenu?*

__________________________ AMOUNT IN KSH

807. The first time that you had sex, did you want to have sex, did you not want to have sex, or were you undecided? / *Je, ulipofanya ngono kwa mara ya kwanza, ulitaka kufanya ngono au haukutaka, au haukuwa umeamua hivyo?*

1=WANTED TO HAVE SEX – *Nilitaka kufanya ngono*
2=DID NOT WANT TO HAVE SEX – *Sikutaka kufanya ngono*
3=WAS UNDECIDED – *Sikuwa nimeamua*

808. I'm going to read a series of statements related to your first sexual experience and I want you to tell me if it relates to you / *Nitakusomea misururu ya semi zinazohusiana na uhusiano wako wa kwanza wa ngono na nataka uniambie kama inahusiana na wewe?* **(READ EACH STATEMENT AND ASK IF THE RESPONDENT AGREES OR DISAGREES)**

	1=AGREE	2=DISAGREE
A. You were physically forced to have your first sexual intercourse / *Ulilazimishwa kimabavu kufanya ngono mara yako ya kwanza*	1	2
B. You were given a gift or money by your partner after the first time you had sex / *ulipewa zawadi ama pesa na mpenzi wako mara ya kwanza ulipofanya ngono*	1	2
C. You had sex the first time to show love to your partner / *Ulifanya ngono mara ya kwanza kumuonyesha mpenzi wako upendo*	1	2
D. You had sex for the first time because you felt obligated because of things your partner had done for you / *Ulifanya ngono mara ya kwanza kwa sababu ulijihisi inakupasa kwa sababu ya vitu mpenzi wako amekufanyia*	1	2
E. You had sex the first time because the person would not take 'no' for an answer / *Ulifanya ngono mara ya kwanza kwa sababu mpenzi wako hakukubali 'la' kuwa jibu*	1	2
F. You/your partner used a condom during your first sex / *Wewe/mpenzi wako mlitumia kondomu mara yako ya kwanza*	1	2
G. You had sex for the first time because you were curious about it / *Ulifanya ngono mara ya kwanza kwa sababu ulikuwa unataka kujua kuihusu*	1	2
H. Your partner hit or beat you to have sex for the first time / *Mpenzi wako alikuchapa au kukupiga ili ufanye ngono mara ya kwanza*	1	2
I. You had sex for the first time because you felt obligated as a partner (spouse or girlfriend) / *Ulifanya ngono mara ya kwanza kwa sababu ulijihisi inakupasa kama mpenzi (mpenzi wako wa ndoa, rafiki wa kike)*	1	2
J. You had sex for the first time because some of your friends pressured you / *Ulifanya ngono mara ya kwanza kwa sababu baadhi ya marafiki zako walikuhimiza*	1	2
K. You had sex for the first time because you thought your other friends were doing it / *Ulifanya ngono mara ya kwanza kwa sababu ulifikiria marafiki zako wengine wanafanya*	1	2
L. You had sex because your partner threatened you / *Ulifanya ngono kwa sababu mpenzi wako alikutishia*	1	2

809. When you had sex for the first time, were you single, engaged to be married, or married? / *Ulipofanya ngono mara ya kwanza, ulikuwa hujaolewa, umeposwa au mumeoana?*

1 = Single
2 = Engaged to be married
3 = Married

810. How many times in the last three months have you had sex? / *Ni mara ngapi kwa miezi mitatu iliyopita umefanya ngono?*

__________________________ NUMBER OF TIMES

811. How many different people have you had sex with in your lifetime? / *Ni watu wangapi tofauti umefanya ngono nao maishani mwako?* **(INCLUDING CURRENT PARTNER)**

_________________________ NUMBER OF PEOPLE

812. How many different people have you had sex with in the last year? / *Ni watu wangapi tofauti umefanya nao ngono katika mwaka uliyopita?* **(INCLUDING CURRENT PARTNER)**

_________________________ NUMBER OF PEOPLE

813. I will read a list of different profiles of people and I want you to tell me if you have ever had sex with such a person. / *Nitakusomea orodha ya watu tofauti na nataka uniambie kama umewahi kufanya ngono na mtu kama huyo.* **(READ THE LIST)**

a. A TOURIST OR FOREIGNER / *Mtalii ama mtu wa nchi nyengine*	1 = NO 2 = YES
b. AN EMPLOYER OF YOURS / *Mwajiri wako*	1 = NO 2 = YES
c. A PERSON 10 OR MORE YEARS OLDER THAN YOU / *Mtu ambaye amekuzidi umri na miaka 10*	1 = NO 2 = YES
d. A TEACHER OF YOURS / *Mwalimu wako yeyote*	1 = NO 2 = YES
e. A PERSON WHO PAID YOU FOR SEX / *Mtu ambaye amekulipa pesa ile mfanye ngono*	1 = NO 2 = YES
f. A PERSON WHO IS MARRIED TO SOMEONE ELSE / *Mume wa mtu mwengine*	1 = NO 2 = YES
g. NONE OF THE ABOVE / *Hamna jibu kati hizi*	1 = NO 2 = YES

Other than the first time you had sex, have you ever had sex because… / *Kando na kufanya ngono kwa mara ya kwanza, umeshawahi fanya ngono kwa sababu ya…*

READ THE LIST. IF RESPONDENT HAS EXPERIENCED SEX IN CONDITION MENTIONED, ASK REMAINDER OF QUESTIONS.

	**814. EVER HAPPENED**	**815. AGE WHEN LAST HAPPENED**	**816. AGE OF PARTNER 91=Don't Know**	**817. RELATION TO PARTNER (CODES)**
A. You were physically forced / *Ulilazimishwa kimabavu*	1= NO 2= YES			
B. You were given a gift or money / *Ulipewa zawadi au pesa*	1= NO 2= YES			
C. Your partner threatened you / *Mpenzi wako alikutishia*	1= NO 2= YES			
D. Your partner would not take 'no' for an answer / *Mpenzi wako hakutaka kukubali 'la' kama jibu*	1= NO 2= YES			
E. Your partner beat you / *Mpenzi wako alikupiga*	1= NO 2= YES			
F. You felt obligated because of what your partner had done for you / *Ulijihisi inakupasa kwa Mpenzi wako kwa sababu ya yale amekufanyia*	1= NO 2= YES			

Relationship to Partner Codes

1 = Husband	5 = Relative	8 = Teacher	12 = Guardian
2 = Boyfriend	6 = Stranger/unknown person	9 = Foreigner/tourist	13 = House guard
3 = Fiancé	7 = Fellow student	10 = Employer	14 = House maid
4 = Friend, acquaintance		11 = Co-worker	15 = Other………

FILTER: INTERVIEWER – CHECK Q808A & Q814A (EVER PHYSICALLY FORCED TO HAVE SEX). IF 'NO' GO TO Q820. IF EVER FORCED TO HAVE SEX, CONTINUE BELOW

818. At the time you were forced to have sex, did you ever tell anyone about it? / *Wakati ulipolazimishwa kufanya ngono, ulimwambia mtu yeyote? (***IF MORE THAN ONE TIME, ASK ABOUT THE MOST RECENT OCCASION)**

1=NO **(GO TO Q820)**
2=YES

819. Who did you tell? / *Ulimwambia nani? (***READ THE LIST)**

1 = HUSBAND/PARTNER/ *Mume wako/mpenzi*	1 = NO	2 = YES
2 = MOTHER/*Mama*	1 = NO	2 = YES
3 = FATHER/*Baba*	1 = NO	2 = YES
4 = MALE FRIEND/*Rafiki mwanamume*	1 = NO	2 = YES
5 = FEMALE FRIEND/Rafiki msichana	1 = NO	2 = YES
6 = BOYFRIEND *mpenzi wa kiume*	1 = NO	2 = YES
7 = EMPLOYER/ *Mwajiri*	1 = NO	2 = YES
8 = TEACHER/ *mwalimu*	1 = NO	2 = YES
9 = NEIGHBOR/ *Jirani*	1 = NO	2 = YES
10= POLICE/*Askari*	1 = NO	2 = YES
11 = OTHER RELATIVES/ *Watu wa jamii*	1 = NO	2 = YES
12 = OTHER NON-RELATIVES/wengine wasio jamaa	1 = NO	2 = YES

820. Have you or your partner ever used a family planning method? / *Je, wewe au mpenzi wako mshawahi tumia njia ya kupanga uzazi?*

1=NO
2=YES **(GO TO Q823)**

821. What is the reason you have not used a family planning method? / *Je, ni kwa sababu gani huja tumia njia za kupanga uzazi?* **(PROBE TWO TIMES, 'ANYTHING ELSE?' CIRCLE ALL THAT APPLY)**

1 = NO PARTNER **(GO TO Q826)**
2 = WANT TO GET PREGNANT
3 = BREASTFEEDING
4 = PARTNER REFUSES
5 = AGAINST RELIGION
6 = DON'T KNOW WHERE TO OBTAIN
7 = AFRAID OF SIDE EFFECTS / INFERTILITY

8 = CLINIC TOO FAR
9 = SUPPLIES NOT ALWAYS AVAILABLE AT CLINIC
96 = OTHER (SPECIFY) ________________________

822. Would you like that you or your partner start using a family planning method? / Je, ungependa wewe au mpenzi wako muanze kutumia njia za kupanga uzazi?

1=NO
2=YES

(GO TO Q826)
823. Which method(s) have you ever used? / *Je, ni njia gani ambazo umewahi kutumia?* (**PROBE TWO TIMES, 'ANYTHING ELSE?' CIRCLE ALL THAT APPLY)**

1 = PILL
2 = IUCD
3 = INJECTABLE/DEPO
4 = MALE CONDOM.
5 = FEMALE CONDOM
6 = VASECTOMY / TUBAL LIGATION
7 = JADELLE /NORPLANT/IMPLANON®
8 = EMERGENCY CONTRACEPTION
9 = NATURAL F.P.
10 = SAFE DAYS
11 = WITHDRAWAL
96=OTHER (SPECIFY)

824. Have you or your partner used a family planning method(s) within the last six months? / *Je, wewe au mpenzi wako mmewahi kutumia njia ya kupanga uzazi miezi sita zilizopita?*

1=NO **(GO TO Q826)**
2=YES

825. Which method(s) have you used within the last six months? /*Je, ni njia gani ambazo umetumia kwa miezi sita zilizopita?* **(PROBE TWO TIMES, 'ANYTHING ELSE?' CIRCLE ALL THAT APPLY)**

1 = PILL
2 = IUCD
3 = INJECTABLE/DEPO
4 = MALE CONDOM.
5 = FEMALE CONDOM
6 = VASECTOMY / TUBAL LIGATION
7 = JADELLE /NORPLANT/IMPLANON®
8 = EMERGENCY CONTRACEPTION
9 = NATURAL F.P.
10 = SAFE DAYS
11 = WITHDRAWAL
12 = OTHER

826. Have you ever used a condom? /*Je, umewahi kutumia kondomu?*

1=NO
2=YES **(GO TO Q828)**

827. Why have you never used a condom? / *Je, kwa nini hujawahi kutumia kondomu?* **(PROBE TWO TIMES, 'ANYTHING ELSE?' CIRCLE ALL THAT APPLY)**

1 = NO RISK OF HIV
2 = PARTNER REFUSED
3 = I REFUSED
4 = I TRUST MY PARTNER
5 = CONDOM WAS NOT AVAILABLE
6 = UNDER INFLUENCE OF ALCOHOL OR DRUGS
7 = AFRAID OF VIOLENCE/THREAT FROM PARTNER
8 = CONDOMS TOO EXPENSIVE

9 = USING OTHER CONTRACEPTIVE
10 = AGAINST RELIGION
11 = NOT FOR MARRIED PEOPLE
96 = OTHER (SPECIFY) _______________________________

(GO TO Q830)

828. On average, how often do/did you use a condom with your regular partner/husband: always, sometimes, or never? /*Je, ni mara ngapi ume/ulitumia kondomu na mpenzi/mume wako: kila wakati, wakati mwengine, hutumii kamwe?*

1 = ALWAYS
2 = SOMETIMES
3 = NEVER

829. The last time you had sex, did you use a condom? */Mara ya mwisho ulipofanya ngono, ulitumia kondomu?*

1=NO
2=YES

830. Have you ever been pregnant? / *Je, umewahi kupata mimba?*

1=NO **(GO TO SECTION 900)**
2=YES

831. How old were you when you had your first pregnancy? / *Je, ulikuwa na miaka mingapi ulipopata mimba ya kwanza?*

_________________________ AGE IN YEARS

832. What was your relationship with the baby's father? / *Je, ulikuwa na uhusiano upi na baba wa mtoto wako?*

1 = HUSBAND
2 = BOYFRIEND
3 = OTHER ACQUAINTANCE
4 = FIANCÉ
5 = STRANGER
96= OTHER (SPECIFY) ____________________________

833. At the time you first became pregnant, did you want to become pregnant then, did you want to be pregnant sooner, did you want to wait until later, or did you not want the pregnancy at all? / *Wakati ulipopata mimba ya kwanza, je, ulitaka kupata mimba wakati huo, ulitaka kupata mimba mapema zaidi, ulitaka kungojea kidogo, au hukutaka kupata mimba kamwe?* **(IF LATER, ASK IF RESPONDENT WANTED TO WAIT A YEAR OR TWO OR LONGER THAN TWO YEARS)**

1 = WANTED TO BE PREGNANT THEN
2 = PREFERRED TO BE PREGNANT SOONER
3 = PREFERRED TO BE PREGNANT ONE OR TWO YEARS LATER
4 = PREFERRED TO BE PREGNANT THREE OR MORE YEARS LATER
5 = DID NOT WANT THE PREGNANCY AT ALL

834. What was your decision regarding your first pregnancy? / *Uwamuzi wako ulikuwa upi kuhusu mimba yako ya kwanza?*

1 = I KEPT IT
2 = I ENDED THE PREGNANCY
3 = I MISCARRIED/HAD A STILLBIRTH
4 = STILL PREGNANT
5 = NO ANSWER

835. In all, how many children have you given birth to? How many boys and how many girls? / *Je, kwa jumla umezaa watoto wangapi? Wavulana ni wangapi na wasichana ni wangapi?*

A. |____|____| TOTAL NUMBER OF CHILDREN
B. |____|____| NUMBER OF BOYS
C. |____|____| NUMBER OF GIRLS

836. Are you pregnant now? / *Je, kwa sasa u mja mzito?*

1=NO
2=YES

FILTER: INTERVIEWER – CHECK Q835A (TOTAL NUMBER OF CHILDREN). IF NO BIRTHS OR ONLY ONE BIRTH, GO TO SECTION 900. IF MORE THAN ONE BIRTH, CONTINUE BELOW

837. I want to talk to you about the most recent birth that you had. How old were you when you last gave birth? / *Nataka kuzungumzia juu ya uzao wako wa hivi karibuni. Je, ulikuwa na miaka mingapi ulipozaa mara ya mwisho?*

_________________________ AGE IN YEARS

838. What was your relationship with the baby's father? / *Je, ulikuwa na uhusiano gani na baba ya mtoto?*

1 = HUSBAND
2 = BOYFRIEND
3 = OTHER ACQUAINTANCE
4 = FIANCÉ
5 = STRANGER
96 = OTHER (SPECIFY) ____________________________

839. At the time you last became pregnant, did you want to become pregnant then, did you want to wait until later, or did you not want the pregnancy at all? / *Wakati uliopata mimba ya kwanza, je, ulitaka kupata mimba wakati huo, ulitaka kupata mimba mapema zaidi, ulitaka kungojea kidogo, au hukutaka kupata mimba kamwe?* **(IF LATER, ASK IF RESPONDENT WANTED TO WAIT A YEAR OR TWO OR LONGER THAN TWO YEARS)**

1 = WANTED TO BE PREGNANT THEN
2 = PREFERRED TO BE PREGNANT SOONER
3 = PREFERRED TO BE PREGNANT ONE OR TWO YEARS LATER
4 = PREFERRED TO BE PREGNANT THREE OR MORE YEARS LATER
5 = DID NOT WANT THE PREGNANCY AT ALL

900. RELATIONSHIPS, MARRIAGE AND VIOLENCE

901. I am going to read a series of statements about marriage and I want you to tell me if you agree or disagree with the statement / *Nitasoma sentensi kadhaa kuhusu ndoa na nataka uniambie kama unakubaliana au hukubaliani na sentensi hizo.*

	1= AGREE	2= DISAGREE	88= DK
A. A wife should be able to refuse her husband sex / *mke anapaswa kuwa na uwezo wa kukataa kufanya ngono na mume wake.*	1	2	88
B. It is OK for a man to cook for his family / *Ni SAWA kwa mwanaume kupikia familia yake*	1	2	88
C. Polygamous marriages are part of your culture so they should continue / *Kuoa wanawake wengi ni sehemu ya mila na desturi zenu kwa hivyo inastahili kuendelea*.	1	2	88
D. It is better if a family arranges a girls' marriage than her choosing herself / *Ni afadhali ikiwa familia itampangia msichana wao kuolewa badala ya kujichagulia mwenyewe*	1	2	88
E. If a man doesn't hit his wife, it means he doesn't love her / *Kama mwanaume hatampiga mkewe, inamaanisha kwamba hampendi*	1	2	88
F. It is a man's right to have sex with his wife whenever he wants / *Ni haki ya mwanaume kufanya ngono na mkewe wakati wo wote anapotaka.*	1	2	88
G. If a young woman in this community were age 25 and unmarried, people would respect her / *Ikiwa mwanamke katika jamii hii atafikisha miaka 25 na hajaolewa, watu wange muheshimu*	1	2	88
H. If a husband and wife disagree on using family planning, the husband's opinion should come first / *Ikiwa mume na mke hawatakubaliana jinsi ya kupanga uzazi, basi, maoni ya mume lazima yaje kwanza*	1	2	88

902. I will read a series of circumstances and I want you to tell me if it is OK for a man to hit or beat his wife in this case: / *Nitasoma baadhi ya hali ya mambo na ningependa uniambie kama ni SAWA kwa mwanaume kumpiga mkewe wakati kama huo.* **(READ THE LIST)**

If she burns the food / *Akiunguza chakula*	*1 = NO*	*2 = YES*
If she argues with him / *Akibishana naye*	*1 = NO*	*2 = YES*
If she goes to the neighbors without telling him / *Akienda kwa majirani bila kumwambia*	*1 = NO*	*2 = YES*
If she refuses to have sex with him / *Akikataa kufanya ngono naye*	*1 = NO*	*2 = YES*
If she neglects the children / *Akikosa kuchunga watoto*	*1 = NO*	*2 = YES*
None of the above / *Hakuna moja ya hiyo*	*1 = NO*	*2 = YES*

Now I would like to ask you questions about some other aspects of a person's life. I know that some of these questions are very personal. Let me assure you that your answers are completely confidential and will not be told to anyone / *Sasa ningependa kukuuliza maswali kuhusu mambo mengine ya maisha yako. Najua ya kwamba maswali mengine ni ya kibinafsi sana. Nina kuhakikishia kwamba majibu yako ni ya siri na hayataambiwa mtu yeyote.*

Has any male ever done any of the following things to you, and, if so, when is the last time it happened: in the last month, in the last 6 months, or in the last year or more than a year ago? / *Mwanamume yeyote ashawahi kufanya vitu vifuatavyo kwako, na, kama ni hivyo, ilifanyika lini mara ya mwisho: kwa mwezi*

uliopita, kwa miezi sita zilizopita, mwaka mmoja uliopita, au zaidi ya mwaka mmoja?

READ THE LIST. IF NO ON Q903, SKIP Q904-905. FOR RELATIONSHIP, ASK ABOUT THE MOST RECENT TIME

Relationship to partner codes

1 = Husband
2 = Boyfriend
3 = Fiancé
4 = Friend, acquaintance
5 = Relative
6 = Neighbor
7 = Fellow student
8 = Teacher
9 = Foreigner/tourist
10 = Employer
11 = Co-worker
12 = Guardian
13 = House guard
14 = House maid
15 = Stranger/unknown person
16 = Other __________

	Q903. Ever Happen	Q904. When Happened				Q905. Relation to Male (see codes)
		IN LAST MONTH	IN LAST 6 MONTHS	IN LAST YEAR	MORE THAN 1 YEAR AGO	
A. Say or do something to humiliate you in front of others? / *Sema au fanya kitu cha kukuaibisha mbele ya wengine?*	1=NO 2=YES	1	2	3	4	
B. Threaten to hurt or harm you or someone close to you? / *Kukutisha au kukuumiza au mtu mwengine karibu nawe?*	1=NO 2=YES	1	2	3	4	
C. Insult you or make you feel bad about yourself? / *Kukutukana au kufanya uhisi vibaya kujihusu?*	1=NO 2=YES	1	2	3	4	
D. Push you, shake you, or throw something at you? / *Kukusukuma, kusukasuka, au kukutupia kitu?*	1=NO 2=YES	1	2	3	4	
E. Slap you? / *Kukupiga kofi?*	1=NO 2=YES	1	2	3	4	
F. Twist your arm or pull your hair? / *Kupinda mkono wako au kuvuta nywele zako?*	1=NO 2=YES	1	2	3	4	
G. Punch you with his fist or something that could hurt you? / *Kukupiga ngumi na mkono wake au kitu ambacho kingeweza kukuumiza?*	1=NO 2=YES	1	2	3	4	
H. Kick you, drag you, or beat you up? / *Kukupiga teke, kuvuruta au kukupiga?*	1=NO 2=YES	1	2	3	4	
I. Try to choke you or burn you on purpose? / *Jaribu kukunyonga au kukuchoma makusudi?*	1=NO 2=YES	1	2	3	4	
J. Threatened to attack you with a knife or other weapon? / *Kukutisha na kisu ama silaha nyingine?*	1=NO 2=YES	1	2	3	4	
K. Attacked you with a weapon? / kukufamia na silaha	1=NO 2=YES	1	2	3	4	
L. Touched you in a sexual way (e.g. kissing, grabbing, or fondling) when you did not want them to? / Kuguswa kwa njia ya kujamiiana (mfano kubusu, kunyakua, au ingia maungoni), wakati wewe hukutaka	1=NO 2=YES	1	2	3	4	
M. Try to have sexual intercourse with you when you did not want to but did not succeed? / Jaribu kufanya ngono na wewe wakati wewe hukutaka lakini hakufanikiwa	1=NO 2=YES	1	2	3	4	

N. Physically forced you to have sexual intercourse even when you did not want to? / *Kukulazimisha kimwili mfanye ngono hata wakati hukutaka*	1=NO<br>2=YES	1	2	3	4
O. Forced you to perform sexual acts when you did not want to? / *Kukulazimisha kufanya vitendo vya ngono wakati hukutaka*	1=NO<br>2=YES	1	2	3	4

FILTER: INTERVIEWER – CHECK Q801 (EVER HAD SEX). IF NEVER HAD SEX (q801=97), GO TO SECTION 1000. IF EVER HAD SEX, CONTINUE BELOW)

906. I am going to read a series of statement about relationships between men and women and I want you to tell me if it applies to your CURRENT OR MOST RECENT PARTNER *Kwanza, nitakuuliza maswahi kuhusu uhusiano wakimaisha kati ya wanaume na wanawake. Tafadhali niambie kama haya yana ambatana na uhusiano wako na mpenzi wako.* ***(IF RESPONDENT HAS NEVER HAD A SEXUAL PARTNER, SELECT N/A)***

	**1=AGREE**	**2=DISAGREE**	**N/A**
A. Your partner is jealous or angry if you talk to someone of the opposite sex / *Mpenzi wako huona wivu ama hasira unapozungumza na mtu wa jinsia nyingine*	1	2	77
B. Your partner frequently accuses you of being unfaithful / *Mpenzi wako hukushuku kuwa wewe si mwaminifu kwake*	1	2	77
C. Your partner permits you to meet your same sex friends / *Mpenzi wako hukuruhusu kukutana na marafiki wa jinsia sawa na yako*	1	2	77
D. Your partner tries to limit your contact with your family / *Mpenzi wako hukuwekea mipaka ya kuwasiliana na familia yako*	1	2	77
E. Your partner insists on knowing where you are at all times / *Mpenzi wako hutaka kujua mahali ulipo kila wakati*	1	2	77
F. Your partner trusts you with money / *Mpenzi wako anakuamini na pesa*	1	2	77

907. In the last six months, has your husband/partner ever withdrawn financial support from you? *Kwa mieze sita zilizopita, mpenzi/mume wako amewahi kuondoa usaidizi wa kifedha kwako?*

1 = NO
2 = YES
9 = DOES NOT PROVIDE FINANCIAL SUPPORT
97 = NO HUSBAND/PARTNER IN THE LAST SIX MONTHS (GO TO Q1000)

908. How many times in the last six months has your husband/partner hit, slapped, kicked or beaten you? *Ni mara ngapi kwa miezi sita zilizopita mume/mpenzi wako amekuchapa, kupiga kofi, kukupiga teke au kukupiga?*

__________________________ NUMBER OF TIMES **(IF '0' GO TO Q911)**

909. The last time you were hit, slapped, kicked or beaten, do you think you deserved it? / *Mara ya mwisho ulipochapwa, pigwa kofi, teke au pigwa wafikiria ulistahili hivyo?*

1 = NO
2 = YES

910. The last time you were hit, slapped, kicked or beaten, had your husband/partner been drinking or using drugs? *Mara ya mwisho ulipochapwa, pigwa kofi, pigwa teke au kupigwa mume/mpenzi wako alikuwa amekunywa au tumia dawa za kulevya?*

1 = NO
2 = YES

911. Have you ever hit, slapped, kicked or beaten your husband/partner? *Umeshawahi kumpiga au kumchapa mume/mpenzi wako?*

1 = NO **(GO TO SECTION 1000)**
2 = YES

912. How many times in the last six months have you hit, slapped, kicked or beaten your husband/partner? *Ni mara ngapi kwa miezi sita iliyopita umewahi mpiga mume/mpenzi wako?*

_________________________ NUMBER OF TIMES

1000. MEMBERSHIP IN OTHER ORGANIZATIONS

Now I am going to ask you about your membership in any organizations that you belonged to prior to today. Please think about all the groups that you are a part of – this would include church or mosque groups, youth groups, school groups, savings groups, sports teams, etc. For each group I am going to ask you to describe a few things about it. / *Sasa nataka nikuulize kuhusu ushirika wako katika mashirika mengine unayoshiriki kabla ya leo. Tafadhali fikiria makundi ye yote ambayo wewe ni mwanachama – hii ni pamoja na makundi ya kanisa, msikiti, makundi ya vijana, makundi ya shule, kuweka akiba, michezo. Nitakuuliza useme mambo mafupi kuhusu kila kundi.*

READ THE LIST. IF NO TO Q1001, SKIP Q1002-1004

		**1001**	**1002**		**1003-1004**	
		Type of group / *Aina ya kundi* (See codes)	All girls or mixed gender / *Ni kundi la wasichana watupu au mchanganyiko*		Main Activities / *Mambo muhimu mnayofanya* (See codes)	
		**No=1 Yes=2**	**Girls Only**	**Mixed Gender**		
A.	SCHOOL GROUP *Kikundi cha shule*		1	2		
B.	YOUTH GROUP *Shirika la vijana*		1	2		
C.	SAVINGS GROUP *Kikundia cha uwekezaji*		1	2		
D.	SPORTS TEAM *Chama cha michezo*		1	2		
E.			1	2		

		**1001**	**1002**		**1003-1004**	
		Type of group / *Aina ya kundi* (See codes)	All girls or mixed gender / *Ni kundi la wasichana watupu au mchanganyiko*		Main Activities / *Mambo muhimu mnayofanya* (See codes)	
	CHURCH/MOSQUE GROUP<br>*Kikundi cha kanisa/msikiti*					
F.	OTHER<br>*NyIngine*		1	2		

MAIN ACTIVITY CODES 1003-1004: **ASK WHAT ARE THE MAIN TOPICS COVERED IN THAT GROUP: / *Mnazungumzia mambo yapi:***

1=HIV
2=REPRODUCTIVE HEALTH
3=FAMILY PLANNING/CONTRACEPTIVES
4=PUBERTY
5=GENDER BASED VIOLENCE
6=CHILDRENS RIGHTS
7=SELF-ESTEEM
8=COMMUNICATION
9=LEADERSHIP
10=SAVINGS
11=FINANCIAL EDUCATION/BUDGETING
12=ENTREPENEURSHIP
13=VOCATIONAL/JOB TRAINING
14=OTHER LIFE SKILLS
15=SPORTS
16=OTHER TOPICS
17=NOT APPLICABLE (**Q1004 ONLY**)

1100. PERCEPTION OF THE INTERVIEW

1101. At any time during the interview, did you feel <u>confused</u>? / *Kuna wakati wowote tukifanya mahojiano ulichanganyikiwa?*

1=NO
2=YES

1102. At any time during the interview, did you feel <u>embarrassed or shy</u>? / *Kuna wakati wowote tukifanya mahojiano uliona aibu?*

1=NO
2=YES

1103. At any time during the interview, did you feel <u>uncomfortable</u>? / *Kuna wakati wowote tukifanya mahojiano haukuona huru?*

1=NO
2=YES

1104. At any time during the interview, did you feel <u>bored</u>? / *Kuna wakati wowote tukifanya mahojiano uliboeka/umechoka*

1=NO
2=YES

1105. Were any of the questions difficult to answer? / *Kuna maswali ambayo yalikuwa ngumu kujibu?*

1=NO (**GO TO 1107)**
2=YES

1106. Which questions? / Maswali gani?

____

____

1107. When you answered these questions, were you very honest, mostly honest or not very honest? *Ulipojibu haya maswali, je ulisema ukweli kabisa,mengi ulisema ukweli ama hukusema ukweli?*

1 = NOT VERY HONEST
2 = MODERATELY HONEST
3 = VERY HONEST

1108. Sometime in the future, an interviewer may return to ask you additional questions. Is this okay with you? / *Wakati ujao ukija kuhojiwa zaidi, itakuwa sawa na wewe?*

1=NO
2=YES

1200. RESPONDENT'S COMMENTS AND INTERVIEWER NOTES

1201. Thank you. I have finished asking my questions. Is there anything else we did not talk about today that you would like to talk about or do you have any questions for me? / *Asante sana! Nimemaliza kuuliza maswali yangu. Je, kuna jambo lo lote ambalo hatujazungumzia leo ambalo ungependa tuzungumzie? Una maswali yo yote ambayo ungependa kuniuliza?*

1=NO
2=YES

1202. Interviewer's Notes / *Ujumbe wa mwenye kuhojiana*

____

____

____

RECORD TIME INTERVIEW ENDS:

Hour: |____|____| Minutes: |____|____|
