# Supplementary material for: Is Working Risky or Protective for Married Adolescent Girls in Urban Slums in Kenya? Understanding the Association between Working Status, Savings and Intimate-Partner Violence
Source: PLoS One. 2016 May 27;11(5):e0155988. doi: 10.1371/journal.pone.0155988 (PMC4883769; doi:10.1371/journal.pone.0155988)
Supplement: S2 File — (PDF) [file pone.0155988.s002.pdf]

Population Council

SERIAL NUMBER

In-depth interview guide for adolescent girls

PART I: IDENTIFICATION	CODES
**LOCATION**<br>1 = THIKA 3 = KISUMU<br>2 = KARIOBANGI 4 = NAKURU	
**VILLAGE OF RESIDENCE**	
**AGE OF RESPONDENT**	
**EVER ATTENDED SCHOOL**<br>0=NO 1=YES	
**YEARS OF EDUCATION COMPLETED**	
**RELIGION:**<br>1=MUSLIM 2=CATHOLIC 3=PROTESTANT 4=OTHER (SPECIFY)	
**OCCUPATION OF RESPONDENT**	
**LIVING ARRAGMENTS OF RESPONDENT**<br>1=BOTH PARENTS 2=MOTHER ONLY 3=FATHER ONLY 4=SPOUSE 5=OTHER RELATIVES 6=NO RELATIVES	
**MARITAL STATUS OF RESPONDENT:**<br>1=NEVER MARRIED 2=MARRIED 3=WIDOWED 4=DIVORCED 5=SEPARATED	
AGE AT FIRST MARRIAGE	
NUMBER OF CHILDREN	
LOCATION OF FIRST INTERVIEW	
NAME OF INTERVIEWER	
DATE OF FIRST INTERVIEW	
DATE OF SECOND INTERVIEW	
DATE OF THIRD INTERVIEW	
SERIAL NUMBER(S) OF TAPES	

SUPERVISOR SIGNATURE AND DATE	
TRANSCRIBER NAME AND DATE	

	Topic	Main questions	Possible probes
		**PART I: WARM UP**	
1.1	**Background**	Tell me about your family and your experiences growing up.	• Where did you grow up?<br>• Tell me about your family.<br>• What was your family's main source of income?<br>• How well-off was your family compared to other families in your community?<br>• Did you consider your family to be rich, poor or in-between? Why?<br>• What were the differences between your family and those who you considered rich/poor/in-between?<br>• Were there any things that you wished you had that your family was not able to afford? What were these? Why?
		**PART II: MONEY AND SAVING**	
2.1	**Income**	Now I'd like to talk about the money that you have and how you spend it. Tell me about the different ways you make money.	• What are your different sources of money?<br>• What are other sources?<br>• How often do you get money from these sources?
2.2	**Expenses**	Tell me about the things you spend your money on.	• What do you do with the money that you get or earn?<br>• What household needs do you spend your money on?<br>• What personal needs do you spend your money on?<br>• What leisure activities do you spend your money on?<br>• Anything else?<br>• Approximately what proportion of your money do you spend on each category: household, personal and leisure activities, or anything else? (Probe: all, three-quarters, half, a third, a quarter, etc)<br>• Do you usually have enough on a weekly basis to cover all your expenses? Why or why not?
2.3	**Saving**	Tell me about any money that you save or put aside.	• Do you ever save money or put money aside?<br>• Why or why not? What are you saving for?<br>• What are some things that make it difficult for you to save? What makes it easy to save?<br>• How often do you save money?<br>• How much do you save on a monthly basis?<br>• What do you use your savings for?
2.4	**Urgent Need for Cash**	Tell me about the options you have when you don't have cash but you need some money urgently.	• Who would you turn to if you needed money urgently? Why? Who else?<br>• Are there some people you cannot turn to? Why?<br>• What are some reasons why you would need money urgently?<br>• Within the last year, what are some emergencies that you have faced that required money? How much money did you need at that time? Where did you get it from? If not, what did you do instead?

	Topic	Main questions	Possible probes
			• Within the last few months, what are some personal items that you have needed to buy? (Probe: clothes, shoes, phone, credit, sanitary towels, etc). How much money did you need at that time? Where did you get it from? If you didn't have the money for a personal item that you really wanted, where would you get it from?<br>• What would you do if you needed money to pay a hospital bill? To pay school fees? To pay for food? Who would you turn to? Why?
		**PART III: RELATIONSHIPS**	
3.1	**Relations between men and women**	Now let's talk about relationships between men and women.	• How do men or boys approach a girl in your community who they are interested in?<br>• How old are girls when they start getting approached by boys or men? How old are the men or boys?<br>• Is there a difference between how a boy approaches a girl compared to an older man? What are the differences?<br>• What are the reasons why some girls start having boyfriends between the ages of 12 and 14?<br>• What are the reasons why girls have boyfriends between the ages of 15 and 17?<br>• What are some things that a boy or man can do to convince a girl he is interested in to become his girlfriend?<br>• What type of man is a girl more likely to have a relationship with if she is from a family that is well-off? Why?<br>• What about when she is from a poor family? Why?<br>• What do girls expect from their boyfriends? Why? (IF NOT MENTIONED): Do they expect anything financially? What? Why?<br>• What do men expect from their girlfriends? Why? Do men expect anything sexual? What else do they expect? Why?
3.2	**Boyfriend**	Have you ever had a boyfriend? By boyfriend, I mean someone who you were emotionally or sexually attracted to when you dated?<br><br>**IF NO: CONTINUE**<br>**IF YES: SKIP TO 3.4**	
3.3	**No Boyfriend**	Why have you never had a boyfriend?	• Any other reason?<br>• Have you ever wanted to have a boyfriend? Why or why not?<br>• Have you been approached by a man who wanted you to become his girlfriend? Tell me about him. How old was he? Was he in school or working? What did he do for a living? Was he single, married, divorced or separated?<br>• How did he approach you and how did you respond? Why?<br>• Did he offer you anything (gifts, money, etc)? Did you accept it? Why or why not?

	Topic	Main questions	Possible probes
		**SKIP TO 3.7**	**SKIP TO 3.7**
3.4	**Current or Most Recent Relationship**	Think about your current or most recent relationship.	• Tell me about your partner. How old was he when you met? Was he in school or working? What did he do for a living?<br>• When you started the relationship was he single, married, divorced or separated?<br>• How old where you when you first met? Where you in school? Which class? If not in school, what were you doing?<br>• Tell me about the relationship. How long did it last? How often did you see each other? How serious was it?
3.5	**Exchange of gifts or money**	Tell me about any gifts that he gave you while you were in the relationship.<br><br>IF MARRIED, ASK ABOUT RELATIONSHIP BEFORE MARRIAGE.<br><br>IF NO GIFTS EXCHANGED IN THIS RELATIONSHIP: Have you ever received gifts from a boyfriend? Tell me about that relationship.	• What types of things did he buy you? How often did he do this?<br>• Did he ever give you money? About how much? How often would he do this?<br>• Did you ever ask him for gifts or money? What did you ask him for? Why? What did he do?<br>• Did you ever borrow money from him? How much did you borrow? What did you need it for? What did he do?<br>• **(IF NOT):** Would you ever borrow money from a boyfriend or ask a boyfriend for money? In which cases would you do so? If not, why not?<br>• Did you ever feel compelled to give him anything or do anything in return for receiving these gifts? Why or why not?<br>• Did he ever ask you to do anything in return after receiving these gifts? What did you do? Why?
3.6	**Intimacy & Contraceptive Use**	Please tell me more about your relationship with your current or most recent partner.	• Did you have any physical contact, such as holding hands, hugging or kissing? Who initiated it? If not, why not?<br>• Did you sleep with him? Who initiated it? How long had you been together when this happened?<br>**(IF NOT, ASK IF EVER HAD SEX)**<br>• The first time you had sex with him; did you want to have sex? Why or why not?<br>• Was there anything that made you feel compelled to have sex with him? What was that?<br>• Did you or your partner use any type of contraceptive that time? Why? Which type?<br>**(IF NO CONTRACEPTIVE):**<br>• Did you want to use a contraceptive? Why did you not? What are the reasons why you did not use a contraceptive (Probe: access, not being able to afford, etc).
3.7	**Other Relationships**	Tell me about other types of relationships that girls have with men who are not their boyfriends. They could be boys their age or older men.	• What are those relationships like?<br>• Where those men in other relationships? What types of relationships? What was their marital status?<br>• What are the reasons why girls have those relationships?<br>• What are the benefits of those relationships?<br>• What are the disadvantages?

	Topic	Main questions	Possible probes
			• Do you know of a girl in one of those relationships? Tell me about it.<br>• Have you ever been in one of those relationships? Tell me about it?
3.8	**Sugar Daddy**	Some girls have relationships with older men who provide for them financially, such as sugar daddies. Tell me about a girl you know who had such a relationship.	• 1. How old was she at the time? Was she in school? Which class?<br>• 2. Tell me about him. What did he do for a living? What was his marital status? How did you/she view his economic status?<br>• 3. What were her reasons for being in the relationship? What were the benefits? What were they disadvantages?<br>• 4. What kinds of things did he give her, provide for her or help her with? (Probe: school expenses, food, shelter/rent, hospital bills, buying clothes, taking care of family members, etc.)<br>• 5. Did she ever feel compelled to give him something or do something in return for receiving this help/money/gifts? What types of things did she do?<br>• 6. Was she sleeping with him? If so, did she or her partner use any type of contraceptive? Why or why not? Which type?<br>• 7. Did he ever use physical violence? In which situations did he do this? What did she do about it?<br>• Have you ever been in a relationship with a sugar daddy? Tell me about it. (**REPEAT PROBES 1-7 ABOVE).**
3.9	**Multiple Partners**	Some girls have more than one boyfriend at the same time. Tell me about a girl you know who has had relationships with more than one man at the same time.	• 1. How old was she at the time? Was she in school? Which class?<br>• 2. Tell me about the different men she was seeing. How many were they? What did they do for a living? What was their marital status?<br>• 3. What were her reasons for being with each man? What benefits did she get from being with more than one man?<br>• 4. How did her relationships with each man differ? How were they similar?<br>• 5. Was she sleeping with any or all of them? Did she and her partners use any type of contraceptive? Why or why not? Which type?<br>• 6. What are some problems she faced by being with more than one man? What were the risks?<br>• Have you ever been in a relationship with more than one man at the same time? Tell me about this. (**REPEAT PROBES 1-6 ABOVE).**
		**PART IV: GENDER-BASED VIOLENCE**	
4.1	**Physical Violence**	Sometimes men use physical violence towards their girlfriends or wives. This includes beating, slapping, kicking, etc.	• Why do you think this happens?<br>• What causes men to do this?<br>• Are there certain situations where a girl or woman deserves this type of violence? Why or why not? Describe the situations.

	Topic	Main questions	Possible probes
			• Which types of men are more likely to do this? (Probe: rich men versus poor men, older versus younger)Why do you think they do this?<br>• What do you think can be done to prevent this from happening to girls? Why?<br>• What do you think can be done to protect girls who have experienced this? Why?
4.2	**Experienced Violence**	Thinking about your own relationships with men. Has any man ever been physically violent with you? Tell me about your relationship with that person.<br><br>IF NEVER EXPERIENCED: Do you know a girl who has experienced this? Tell me about that relationship.	**CHECK IF IT'S A PARTNER THAT RESPONDENT HAS ALREADY DESCRIBED ABOVE**<br>**(IF DIFFERENT PARTNER):**<br>• Tell me about that man. What was your relationship with him? How old was he when you met? Was he in school or working? What did he do for a living? What was his marital status?<br>• How old where you when you first met? Where you in school? Which class? If not in school, what were you doing?<br>• Tell me about any gifts that he gave you before this happened.<br>• Did you ever feel compelled to give him anything or do anything in return for receiving these gifts? Why or why not?<br>**(ALL):**<br>• Tell me about the first time he was violent with you.How old were you at the time? How long had you been in the relationship/how long had you known him? Was he supporting you financially at that time? How?<br>• When he got violent, what did he do? Why do you think he got violent? Did you expect it to happen? Why or why not?<br>• How did you react to this? What did you do? Did you think about leaving him? Why? If not, why not?<br>• Did it ever happen again? How often did it happen?<br>• Did you ever tell anyone about it or seek help after it happened? Why or why not? Who did you tell (Probe: parent/guardian, sibling, friend, doctor, teacher)?
4.3	**Sexual Violence**	Sometimes, men pressure or force their girlfriends or wives to have sex or to do sexual acts when they do not want to.	• Why do you think this happens?<br>• What causes men to do this?<br>• Are there certain situations where a girl or woman deserves this type of violence? Why or why not? Describe the situations.<br>• Which types of men are more likely to do this? (Probe: rich men versus poor men, older versus younger)Why do you think they do this?<br>• What do you think can be done to prevent this from happening to girls? Why?<br>• What do you think can be done to protect girls who have experienced this? Why?
4.4	**Experienced Sexual Violence**	Thinking about your own relationships with men. Has any man ever	**CHECK IF IT'S A PARTNER THAT RESPONDENT HAS ALREADY DESCRIBED ABOVE**

	Topic	Main questions	Possible probes
		pressured or forced you to have sex or do sexual acts when you did not want to?<br><br>IF NEVER EXPERIENCED: Do you know a girl who has experienced this? Tell me about that relationship.	**(IF DIFFERENT PARTNER):**<br>• Tell me about that man. What was your relationship with him? How old was he when you met? Was he in school or working? What did he do for a living? What was his marital status?<br>• How old where you when you first met? Where you in school? Which class? If not in school, what were you doing?<br>• Tell me about any gifts that he gave you before this happened.<br>• Did you ever feel compelled to give him anything or do anything in return for receiving these gifts? Why or why not?<br>**(ALL):**<br>• Tell me about the first time he pressured or forced you to do something sexually that you did not want to. How old were you at the time? How long had you been in the relationship/how long had you known him? Was he supporting you financially at that time? How?<br>• What did he pressure or force you to do? Why do you think he did this? Did you expect it to happen? Why or why not?<br>• How did you react to this? What did you do? Did you think about leaving him? Why? If not, why not?<br>• Did it ever happen again? How often did it happen?<br>• Did you ever tell anyone about it or seek help after it happened? Why or why not? Who did you tell (Probe: parent/guardian, sibling, friend, doctor, teacher)?
		**PART V: ECONOMIC ASSETS & SEXUAL RELATIONSHIPS**	
5.1	**Increased Economic Assets**	Imagine a 16-year old girl who has never had any money on her own. She gets a new business idea and starts making money.	• How would she benefit from this money? How would it improve her life? What new things would she be able to do?<br>• What are some risks or problems that she would face because of this income? (Probe: any risk of being teased, being harassed, experiencing violence?)<br>• What would help to reduce these risks? (Probe: friends, having a mentor, self-esteem, etc).<br>• How would this income affect her relationships with boys or men?<br>• How would it affect how she is viewed and treated by boys or men?<br>• Would it affect whether or not she gets into relationships with boys or men? Why or why not?<br>• What type of man would she be most likely to get into a relationship with?<br>• Would it affect whether or not she has sex with her boyfriend? Why or why not?<br>• If she was not ready to have sex, how would the income affect her ability to refuse?
5.2	**Reduced Economic Assets**	Now, imagine that the same girl lost her business	• What are some of the things that she would do to meet her needs, now that she does not have the income?

	Topic	Main questions	Possible probes
		and was no longer earning any income.	• What are some of the risks or problems she would face due to her lack of income?<br>• How would the lack of income affect her relationships with boys or men? How would it affect whether or not she has sex? How would it affect her ability to refuse sex if she does not want to?<br>• How would things be different for a girl who has a family to support her financially, compared to a girl with no parents who is supporting her younger siblings? (Probe: risks she would face, relationships with men, sex and ability to refuse sex).
5.3	**Savings as Economic Asset**	Now, imagine that the girl lost her business, but she had some savings in a savings account.	• How would the savings account help her in this situation?<br>• How would it reduce some of the risks or problems she is facing because of her lack of income?<br>• How would the saving affect her relationships with boys or men? How would it affect whether or not she has sex? How would it affect her ability to refuse sex if she does not want to?<br>• Are there any risks she would face because of having the savings? (Probe: any risk of being teased, being harassed, experiencing violence?)<br>• What would help to reduce these risks? (Probe: friends, having a mentor, self-esteem, etc).
5.4	**Used Own Savings**	Thinking about your own experiences, have you ever used your savings to help you during an emergency?<br><br>IF NEVER, ASK: Have you ever been in a situation where having savings would have helped you during an emergency?	• What was the situation? How much money did you need? What did you need it for?<br>• What other options did/would you have for getting this money? Who else could/would you have turned to? What problems/risks did/would you face if you did NOT get this money?<br>• How did/would the savings help?
		**WRAP UP**	
		• We have reached the end of the interview. Before we finish, is there anything else that you would like to add regarding anything that we have discussed?	
		• If you wanted more information on the topics we discussed today, do you have somewhere to go?	

Thank you. Thank you for sharing your thoughts and experiences with me. As we mentioned, this information will be used to improve programs for young people in Kenya. If you want to discuss anything further with a professional, I can give you a phone number for someone who will assist you in getting the help you need.

Interviewer observations (use back of page if necessary):

……………………………………………………………………………………………..

……………………………………………………………………………………………………..

……………………………………………………………………………………………………..
