# Supplementary material for: Is Working Risky or Protective for Married Adolescent Girls in Urban Slums in Kenya? Understanding the Association between Working Status, Savings and Intimate-Partner Violence
Source: PLoS One. 2016 May 27;11(5):e0155988. doi: 10.1371/journal.pone.0155988 (PMC4883769; doi:10.1371/journal.pone.0155988)
Supplement: S3 File — (PDF) [file pone.0155988.s003.pdf]

Population Council

SERIAL NUMBER

In-depth interview guide for young men

PART I: IDENTIFICATION	CODES
**LOCATION**<br>1 = THIKA 3 = KISUMU<br>2 = KARIOBANGI 4 = NAKURU	
**VILLAGE OF RESIDENCE**	
**AGE OF RESPONDENT**	
**EVER ATTENDED SCHOOL**<br>0=NO 1=YES	
**YEARS OF EDUCATION COMPLETED**	
**RELIGION:**<br>1=MUSLIM 2=CATHOLIC 3=PROTESTANT 4=OTHER (SPECIFY) ………………………………………………	
**OCCUPATION OF RESPONDENT**	
**LIVING ARRAGMENTS OF RESPONDENT**<br>1=BOTH PARENTS 2=MOTHER ONLY 3=FATHER ONLY 4=SPOUSE 5=OTHER RELATIVES 6=NO RELATIVES	
**MARITAL STATUS OF RESPONDENT:**<br>1=NEVER MARRIED 2=MARRIED 3=WIDOWED 4=DIVORCED 5=SEPARATED	
AGE AT FIRST MARRIAGE	
NUMBER OF CHILDREN	
LOCATION OF FIRST INTERVIEW	
NAME OF INTERVIEWER	
DATE OF FIRST INTERVIEW	
DATE OF SECOND INTERVIEW	
DATE OF THIRD INTERVIEW	
SERIAL NUMBER(S) OF TAPES	

SUPERVISOR SIGNATURE AND DATE	
TRANSCRIBER NAME AND DATE	

	Topic	Main questions	Possible probes
		**PART I: WARM UP**	
1.1	**Background**	Tell me about your family and your experiences growing up.	• Where did you grow up?<br>• Tell me about your family.<br>• What was your family's main source of income?<br>• How well-off was your family compared to other families in your community?<br>• Did you consider your family to be rich, poor or in-between? Why?<br>• What were the differences between your family and those who you considered rich/poor/in-between?<br>• Were there any things that you wished you had that your family was not able to afford? What were these? Why?
		**PART II A: OWN MONEY AND SAVING**	
2.1	**Income**	Now I'd like to talk about the money that you have and how you spend it. Tell me about the different ways you make money.	• What are your different sources of money?<br>• What are other sources?<br>• How often do you get money from these sources?
2.2	**Expenses**	Tell me about the things you spend your money on.	• What do you do with the money that you get or earn?<br>• What household needs do you spend your money on?<br>• What personal needs do you spend your money on?<br>• What leisure activities do you spend your money on?<br>• Anything else?<br>• Approximately what proportion of your money do you spend on each category: household, personal and leisure activities, or anything else? (Probe: all, three-quarters, half, a third, a quarter, etc)
2.3	**Saving**	Tell me about any money that you save or put aside.	• Do you ever save money or put money aside?<br>• Why or why not? What are you saving for?<br>• What are some things that make it difficult for you to save? What makes it easy to save?<br>• How often do you save money?<br>• How much do you save on a monthly basis?<br>• What do you use your savings for?
		**PART II B: GIRLS' MONEY AND SAVING**	
2.4		Now I would like you to think about young women in your community, particularly girls between the ages of 15 and 19. Tell me how you think they get money.	• What are their main sources of income?<br>• For girls who have jobs, what are the most common types of jobs that they do?<br>• For girls who do not have jobs, where do they usually get money from?<br>• Where do school girls usually get money from?<br>• If a young woman has an emergency or an urgent need, who would she turn to for the money? Why?<br>• Would she turn to a man for money? Why? What types of girls would turn to men for money?

	Topic	Main questions	Possible probes
		**PART III: RELATIONSHIPS**	
3.1	**Relations between men and women**	Now let's talk about relationships between men and women.	• How do men or boys approach a girl in your community who they are interested in?<br>• How old are girls when they start getting approached by boys or men? How old are the men or boys?<br>• Is there a difference between how a boy approaches a girl compared to an older man? What are the differences?<br>• What are the reasons why some girls start having boyfriends between the ages of 12 and 14?<br>• What are the reasons why girls have boyfriends between the ages of 15 and 17?<br>• What are some things that a boy or man can do to convince a girl he is interested in to become his girlfriend?<br>• What type of man is a girl more likely to have a relationship with if she is earning her own income or she has her money of her own? Why?<br>• What about when she does not have any money of her own? Why?<br>• What do girls expect from their boyfriends? Why? (IF NOT MENTIONED): Do they expect anything financially? What? Why?<br>• What do men expect from their girlfriends? Why? Do men expect anything sexual? What else do they expect? Why?
3.2	**Current or Most Recent Relationship**	Think about your current or most recent relationship.	• Tell me about the girl. How old was she when you met? Was she in school or working? If working, what did she do for a living? What was her marital status?<br>• When you started the relationship were you single, married, divorced or separated?<br>• How old where you when you first met?Where you working at the time? What kind of work were you doing? If not, what were you doing?<br>• Tell me about the relationship. How long did it last? How often did you see each other? How serious was it?
3.5	**Exchange of gifts or money**	Tell me about any gifts that you gave you while you were in the relationship.<br><br>IF MARRIED, ASK ABOUT RELATIONSHIP BEFORE MARRIAGE.<br><br>IF NO GIFTS EXCHANGED IN THIS RELATIONSHIP: Have you ever given gifts to a girlfriend? Tell me about that relationship.	• What types of things did you buy her? How often did you do this? Why did you do this?<br>• Did you ever give her money? About how much? How often would you do this? Did you expect anything from her because of this?<br>• Did she ever ask you for gifts or money? What did she ask you for? Why did she ask for it? What did you do?<br>• Did she ever borrow money from you? How much did she borrow? What did she need it for? What did you do? Did you expect her to pay it back? Why or why not? Did she ever pay it back? Why or why not?<br>• (IF NOT): Would you ever lend money to a girlfriend if she asked for it? In which cases would you do so? If not, why not?

	Topic	Main questions	Possible probes
			• How did you feel after you gave her the gifts or money? How did she behave? Did anything change after you gave her the gifts or money? What changed? Why?<br>• Did she do anything in return for the money or gifts she received? What did she do? Why?<br>• Did she do anything to show you how much she appreciated getting the gifts or money? What did she do? Why do you think she did this?<br>• Did you ask her to do anything in return after she received these gifts? What did ask her to do? Why?
3.6	**Intimacy & Contraceptive Use**	Please tell me more about your relationship with your current or most recent partner.	• Did you have any physical contact, such as holding hands, hugging or kissing? Who initiated it? If not, why not?<br>• When you first met her, did you expect her to sleep with you? Why or why not?<br>• Did you sleep with her? Who initiated it? How long had you been together when this happened?<br>• The first time you had sex with her; did she want to have sex? Why or why not?<br>• Did you or your partner use any type of contraceptive that time? Why? Which type?<br>**(IF NO CONTRACEPTIVE):**<br>• Did you want to use a contraceptive? Why did you not? What are the reasons why you did not use a contraceptive (Probe: did not have any, do not like it, etc).
3.7	**Other Relationships**	Tell me about other types of relationships that girls have with men who are not their boyfriends. They could be boys their age or older men.	• What are those relationships like?<br>• Do those men tend to be in other relationships? What types of relationships? What about relationships with married men?<br>• What are the reasons why girls have those relationships?<br>• What are the benefits of those relationships?<br>• What are the disadvantages?<br>• Do you know of a girl in one of those relationships? Tell me about it.<br>• Have you ever been in one of those relationships with a girl? Tell me about it.How did the relationship start? What were the benefits of this type of relationship? What were the disadvantages? What purpose did the relationship serve?
3.8	**Sugar Daddy**	Some girls have relationships with older men who provide for them financially, such as sugar daddies. Tell me about a girl you know who had such a relationship.	• 1. How old was she at the time? Was she in school? Which class?<br>• 2. Tell me about the man. What did he do for a living? What was his marital status? How did you/she view his economic status?<br>• 3. What were her reasons for being in the relationship? What were the benefits? What were they disadvantages?<br>• 4. What kinds of things did he give her, provide for her or help her with? (Probe: school expenses, food, shelter/rent, hospital bills, buying clothes, taking care of family members, etc.)

	Topic	Main questions	Possible probes
			• 5. Did she ever feel compelled to give him something or do something in return for receiving this help/money/gifts? What types of things did she do?<br>• 6. Was she sleeping with him? If so, did she or her partner use any type of contraceptive? Why or why not?<br>• 7. Did he ever use physical violence? In which situations did he do this? What did she do about it?<br>• Have you ever been in this type of a relationship with a girl? Tell me about it. (**REPEAT PROBES 1-7 ABOVE).**
3.9	**Multiple Partners**	Some girls have more than one boyfriend at the same time. Tell me about a girl you know who has had relationships with more than one man at the same time.	• 1. How old was she at the time? Was she in school? Which class?<br>• 2. Tell me about the different men she was seeing. How many were they? What did they do for a living? What was their marital status?<br>• 3. What were her reasons for being with each man? What benefits did she get from being with more than one man?<br>• 4. How did her relationships with each man differ? How were they similar?<br>• 5. Was she sleeping with any or all of them? Did she and her partners use any type of contraceptive? Why or why not?<br>• 6. What are some problems she faced by being with more than one man? What were the risks?<br>• Have you ever been in a relationship with more than one man at the same time? Tell me about this. (**REPEAT PROBES 1-6 ABOVE).**
		**PART IV: GENDER-BASED VIOLENCE**	
4.1	**Physical Violence**	Sometimes men use physical violence towards their girlfriends or wives. This includes beating, slapping, kicking, etc.	• Why do you think this happens?<br>• What causes men to do this?<br>• Are there certain situations where a girl or woman deserves this type of violence? Why or why not? Describe the situations.<br>• What types of girls are more likely to experience this? Why?<br>• Which types of men are more likely to do this? (Probe: rich men versus poor men, older versus younger)Why do you think they do this?<br>• What do you think can be done to prevent this from happening to girls? Why?<br>• What do you think can be done to protect girls who have experienced this? Why?
4.2	**Sexual Violence**	Sometimes, men pressure or force their girlfriends or wives to have sex or to do sexual acts when they do not want to.	• Why do you think this happens?<br>• What causes men to do this?<br>• Are there certain situations where a girl or woman deserves this type of violence? Why or why not? Describe the situations.<br>• What types of girls are more likely to experience this? Why?

	Topic	Main questions	Possible probes
			• Which types of men are more likely to do this? (Probe: rich men versus poor men, older versus younger)Why do you think they do this?<br>• What do you think can be done to prevent this from happening to girls? Why?<br>• What do you think can be done to protect girls who have experienced this? Why?
		**PART V: ECONOMIC ASSETS & SEXUAL RELATIONSHIPS**	
5.1	**Increased Economic Assets**	Imagine a 16-year old girl who has never had any money on her own. She gets a new business idea and starts making money.	• How would she benefit from this money? How would it improve her life? What new things would she be able to do?<br>• What are some risks or problems that she would face because of this income? (Probe: any risk of being teased, being harassed, experiencing violence?)<br>• What would help to reduce these risks? (Probe: friends, having a mentor, self-esteem, etc).<br>• How would this income affect her relationships with boys or men?<br>• How would it affect how she is viewed and treated by boys or men?<br>• Would it affect whether or not she gets into relationships with boys or men? Why or why not?<br>• What type of man would she be most likely to get into a relationship with?<br>• Would it affect whether or not she has sex with her boyfriend? Why or why not?<br>• If she was not ready to have sex, how would the income affect her ability to refuse?
5.2	**Reduced Economic Assets**	Now, imagine that the same girl lost her business and was no longer earning any income.	• What are some of the things that she would do to meet her needs, now that she does not have the income?<br>• What are some of the risks or problems she would face due to her lack of income?<br>• How would the lack of income affect her relationships with boys or men? How would it affect whether or not she has sex? How would it affect her ability to refuse sex if she does not want to?<br>• How would things be different for a girl who has a family to support her financially, compared to a girl with no parents who is supporting her younger siblings? (Probe: risks she would face, relationships with men, sex and ability to refuse sex).
5.3	**Savings as Economic Asset**	Now, imagine that the girl lost her business, but she had some savings in a savings account.	• How would the savings account help her in this situation?<br>• How would it reduce some of the risks or problems she is facing because of her lack of income?<br>• How would the saving affect her relationships with boys or men? How would it affect whether or not she has sex? How would it affect her ability to refuse sex if she does not want to?

	Topic	Main questions	Possible probes
			• Are there any risks she would face because of having the savings? (Probe: any risk of being teased, being harassed, experiencing violence?)<br>• What would help to reduce these risks? (Probe: friends, having a mentor, self-esteem, etc).
		**WRAP UP**	
		• We have reached the end of the interview. Before we finish, is there anything else that you would like to add regarding anything that we have discussed?	
		• If you wanted more information on the topics we discussed today, do you have somewhere to go?	

Thank you. Thank you for sharing your thoughts and experiences with me. As we mentioned, this information will be used to improve programs for young people in Kenya. If you want to discuss anything further with a professional, I can give you a phone number for someone who will assist you in getting the help you need.

Interviewer observations (use back of page if necessary):

……………………………………………………………………………………………………..

……………………………………………………………………………………………………..

……………………………………………………………………………………………………..
